# Heterogeneous nanoscopic lipid diffusion in the live cell membrane and its dependency on cholesterol

**DOI:** 10.1016/j.bpj.2022.07.008

**Published:** 2022-07-16

**Authors:** Yu-Jo Chai, Ching-Ya Cheng, Yi-Hung Liao, Chih-Hsiang Lin, Chia-Lung Hsieh

**Affiliations:** 1 Institute of Atomic and Molecular Sciences (IAMS), Academia Sinica, Taipei, Taiwan

## Abstract

Cholesterol plays a unique role in the regulation of membrane organization and dynamics by modulating the membrane phase transition at the nanoscale. Unfortunately, due to their small sizes and dynamic nature, the effects of cholesterol-mediated membrane nanodomains on membrane dynamics remain elusive. Here, using ultrahigh-speed single-molecule tracking with advanced optical microscope techniques, we investigate the diffusive motion of single phospholipids in the live cell plasma membrane at the nanoscale and its dependency on the cholesterol concentration. We find that both saturated and unsaturated phospholipids undergo anomalous subdiffusion on the length scale of 10–100 nm. The diffusion characteristics exhibit considerable variations in space and in time, indicating that the nanoscopic lipid diffusion is highly heterogeneous. Importantly, through the statistical analysis, apparent dual-mobility subdiffusion is observed from the mixed diffusion behaviors. The measured subdiffusion agrees well with the hop diffusion model that represents a diffuser moving in a compartmentalized membrane created by the cytoskeleton meshwork. Cholesterol depletion diminishes the lipid mobility with an apparently smaller compartment size and a stronger confinement strength. Similar results are measured with temperature reduction, suggesting that the more heterogeneous and restricted diffusion is connected to the nanoscopic membrane phase transition. Our conclusion supports the model that cholesterol depletion induces the formation of gel-phase, solid-like membrane nanodomains. These nanodomains undergo restricted diffusion and act as diffusion obstacles to the membrane molecules that are excluded from the nanodomains. This work provides the experimental evidence that the nanoscopic lipid diffusion in the cell plasma membrane is heterogeneous and sensitive to the cholesterol concentration and temperature, shedding new light on the regulation mechanisms of nanoscopic membrane dynamics.

## Significance

Biological membrane functions are closely regulated by membrane organization and dynamics. Phase separation has been recognized as an important mechanism for modulating membrane structures and dynamics. Here, using advanced optical microscopy and single-molecule techniques, we unveil the complex diffusion of phospholipids in the plasma membrane of live cells at ultrahigh spatiotemporal resolutions. We find that both saturated and unsaturated phospholipids undergo restricted diffusion in the plasma membrane on the length scale of tens of nanometers. The restriction properties are sensitive to the cholesterol concentration and temperature, suggesting that membrane phase separation plays a significant role in modulating nanoscale membrane dynamics. Our results show that cholesterol improves the long-range molecular diffusion, most likely by preventing the formation of gel-phase membrane nanodomains.

## Introduction

Cell plasma membrane is a complex fluidic system composed of a great diversity of lipids and proteins. The interactions between individual molecules give rise to the heterogeneous membrane organizations, including protein clusters (1, 2, 3) and cholesterol-dependent nanodomains (e.g., lipid rafts) (4,5). In addition to the spatial heterogeneity, these membrane structures are dynamic, occurring over a wide range of timescale (6,7). The membrane organization constantly affects the motion of membrane molecules, which modulates the membrane dynamics and functions (8,9).

Investigations of membrane dynamics, both experimentally and computationally, often observe anomalous diffusion, a diffusive motion that deviates from simple free diffusion (10, 11, 12). By measuring the motion of single plasma membrane molecule through single-particle tracking (SPT) at a high speed (25-μs time resolution), it was found that the lipids and proteins undergo hop diffusion in a compartmentalized membrane created by the cytoskeleton meshwork underneath the membrane (13, 14, 15, 16). The typical size of the membrane compartments of mammalian cells is tens of nanometers (13). Nanoscale membrane dynamics and lipid interactions have also been characterized by fluorescence correlation spectroscopy (FCS) measurement with superresolution stimulated emission depletion (STED) (17, 18, 19). With a spatial resolution down to 40 nm, compartmentalized phospholipid diffusion by actin cytoskeleton was observed (18). At a smaller length scale (i.e., within the mesh compartments of tens of nanometers), numerical computation studies report anomalous subdiffusion of membrane proteins induced by molecular crowding (20, 21, 22).

Cholesterol plays a unique role in the formation of dynamic membrane nanodomains, known as lipid rafts, whose size ranges from 10 to 200 nm (5,23). Lipid rafts are enriched in (glyco)sphingolipids and cholesterol and are thought to act as functional local platforms in the plasma membrane that recruit raft-associating molecules into the domain (24,25). Previous study reported the transient confinements of sphingolipids and GPI-anchored proteins in the isolated cholesterol-dependent nanodomains (8). Using STED-FCS, anomalous subdiffusion of sphingomyelin and GPI-anchored protein was observed at 20- to 40-nm length scale by cholesterol-mediated complexes (19).

The effects of cholesterol in membrane organization and dynamics have been extensively investigated in the model systems and in the live cells (26, 27, 28, 29). In the study of phase-separated model membrane, the addition of a proper amount of cholesterol into the binary liquid-gel lipid bilayers transforms the gel phase into a more fluidic liquid-disordered (Lo) phase in which the lipid mobility is significantly enhanced (30, 31, 32, 33). Meanwhile, the lipid mobility of the liquid-disordered (Ld) phase is nearly unchanged by the incorporation of cholesterol (33,34). Depletion of cholesterol in the cell plasma membrane induces solid-like regions that act as diffusion obstacles to the lipids and proteins (35,36). Cholesterol depletion was also found to interrupt the regulation of membrane signaling, which could be associated with the disruption of lipid rafts (37, 38, 39).

To understand how the incorporation of cholesterol affects the membrane dynamics and functions, it would be valuable to investigate the effects of cholesterol at the length scale of 10–100 nm, which corresponds to the size of protein clusters, lipid rafts, and membrane compartments. Such a length scale is difficult to reach by conventional optical microscopy techniques due to the diffraction limit of light. Moreover, a high temporal resolution (sub-milliseconds to microseconds) is simultaneously needed when attempting to resolve the dynamics that occur below 100 nm because of the fluidic nature of the membrane.

Advancements in optical microscope techniques have enabled the measurements of membrane dynamics at high spatiotemporal resolutions. The superresolution fluorescence microscope technique, STED-FCS, measures the diffusion characteristics of a target molecule with a spatial resolution down to 40 nm (17, 18, 19). By varying the size of the detection area, the molecular diffusion mode can be determined (40,41). SPT is another promising technique that measures the continuous diffusion trajectory of a single target molecule labeled by an optical probe. The SPT is especially powerful in characterizing complex diffusion where the motion is heterogeneous in space and in time (42,43). For high-speed scattering-based SPT, metallic nanoparticles with sizes of 20–50 nm are commonly used as the optical probes (14,44). A recent study showed that, although the size of the particle is large compared with the target molecule, the hydrodynamic loading of the particle does not affect the measured diffusion if the labeling is monovalent and oriented (45). With a gold particle as the imaging probe, recent advance on interferometric scattering (iSCAT) microscopy enhances the signal-to-noise ratio (SNR) and offers the opportunity to measure the motion of a single membrane protein with a spatial precision of a few nanometers in three dimensions (3D) at a time resolution up to ∼15 μs (44).

In this work, we employ advanced scattering-based interference optical microscopy to perform high-resolution single phospholipid tracking in the live cell plasma membrane. Two probe lipids are studied: the saturated lipid 1,2-distearoyl-sn-glycero-3-phosphoethanolamine (DSPE) and the unsaturated phospholipid 1,2-dioleoyl-sn-glycero-3-phosphoethanolamine (DOPE), representing the raft-associated and non-raft lipids, respectively. We find that both lipids undergo anomalous subdiffusion in the 0.1- to 10-ms timescale and 10- to 100-nm length scale. Transient diffusion analyses show that the nanoscopic lipid diffusion is heterogeneous in space and in time. Importantly, dual mobility is observed for both lipids. We characterize the subdiffusion of the individual two mobilities based on the model of hop diffusion. We further investigate the effects of phase separation on the membrane dynamics by manipulating the cholesterol concentration and by changing the sample temperature. Our data show that the membrane compartmentalization experienced by the phospholipid is sensitive to the cholesterol concentration and temperature. This indicates that the interactions between the membrane molecules and the cytoskeleton meshwork are strongly affected by the phase separation condition of the membrane, possibly through the formation of membrane nanodomains.

## Materials and methods

### Materials

Non-fluorescent lipids, 1-palmitoyl-2-oleoyl-glycero-3-phosphocholine (POPC), 1,2-dioleoyl-sn-glycero-3-phosphocholine (DOPC), 1,2-distearoyl-sn-glycero-3-phosphoethanolamine-N-[biotinyl(polyethylene glycol)-2000] (DSPE-PEG2000-biotin), 1,2-dioleoyl-sn-glycero-3-phosphoethanolamine-N-[biotinyl(polyethylene glycol)-2000] (DOPE-PEG2000-biotin), 1,2-diphytanoyl-sn-glycero-3-phosphocholine (DiPhyPC), 1,2-dipalmitoyl-sn-glycero-3-phosphocholine (DPPC), and cholesterol, were purchased from Avanti Polar Lipids (AL, USA). Note that DOPE-PEG2000-biotin was a custom-made product. Fluorescent lipid dioctadecyloxacarbocyanine (DiO), methyl-β-cyclodextrin (mβCD), and CK-666 were purchased from Sigma-Aldrich (MO, USA). Bare gold nanoparticle colloidal (AuNP, 30 nm) was purchased from BBI Solutions (Cardiff, UK). Heterobifunctional monothiolalkane PEG tethers, monothiolalkane(C11)PEG3-OH, monothiolalkane(C11)PEG3-COOH, and biotinylated alkanePEG thiol (C_31_H_58_N_4_O_6_S_2_; molecular weight 646.95), were obtained from Sensopath Technologies (MT, USA). The buffer used in SPT was purchased from GE Healthcare Life Sciences (Buckinghamshire, UK). HEPES was obtained from Sigma-Aldrich (MO, USA).

### Functionalization of AuNPs and rAv conjugation

The protocols for surface functionalization of AuNP and its conjugation to rhizavidin (rAv) were reported previously by our group (45). In brief, the surface of bare 30-nm AuNP (BBI) was biotinylated with monothiolalkane (C11) PEG3-OH, monothiolalkane(C11)PEG3-COOH, and biotinylated alkane polyethylene glycol (PEG) thiol in a molar ratio of 1:1:0.05–0.1. After removing the excess amount of the PEG tethers by buffer exchange using centrifugation, rAv was added into the solution of freshly functionalized AuNP solution and incubated for 3 h at 25°C under 500 rpm orbital agitation. Buffer exchange was applied to remove the excess amount of rAv using centrifugation. PEG 20,000 was added to the rAv-AuNP colloidal solution at 0.1% to finalize the rAv conjugation.

### Cell culture

The PtK2 cells were maintained in minimum essential medium (HyClone) supplemented with 10% fetal bovine serum (HyClone) and 100 U/mL penicillin and 10 μg/mL streptomycin (Hyclone). Then 5 × 10^5^ PtK2 cells per dish were seeded in 6-cm culture dishes (Corning) and incubated for 24 h at 37°C while supplemented with 5% CO_2_. The PtK2 cell is chosen for our investigation because its cell morphology is relatively flat when cultured on cover glass, and thus it is easier for microscope imaging and SPT.

### Introduction of biotinylated lipids into the plasma membrane through membrane fusion

The biotinylated probe lipids were introduced to the plasma membrane of live cells following a slightly modified protocol reported previously (46). Briefly, the biotinylated lipid was mixed in 1:1 molar ratio with POPC dissolved in chloroform. The chloroform was removed by applying a gentle flow of nitrogen gas inside the vial. The vial was placed under vacuum for at least 1 h to further diminish the chloroform residuals. The dried lipid film was hydrated in 10 mM HEPES buffer (pH 7) at 60°C at a final concentration of 2 mg/mL. The lipid solution was vortexed at room temperature and then bath sonicated for 20 min at 60°C, creating fusogenic liposomes. The liposome solution was stored at 4°C for no more than 2 days before use. Fusogenic liposomes were diluted by pre-warmed HEPES-buffered serum-free culture medium at a final concentration of 20 μg/mL. The cells were washed with the HEPES-buffered medium twice. The medium was then replaced with equal volume of diluted fusogenic liposome solution and incubated at 37°C for 10 min. The membrane fusion was finalized by washing the cells with HEPES-buffered medium three times to remove the excess liposomes.

### AuNP labeling

We added 10 μL of 10-fold-concentrated rAv-AuNP (via centrifugation) into the buffered cell medium for ∼2 min. The unbound rAv-AuNPs were removed by repeated medium exchanges. Microscope imaging and SPT were performed immediately after AuNP labeling. The specificity of AuNP labeling to the biotinylated lipid was examined by the control experiments where either the biotinylated lipids or the rAv is absent. In these control experiments, the number of AuNPs on the cell surface is reduced by at least a factor of 10, indicating that the rAv-AuNP was able to attach to the biotinylated lipids with high specificity.

### Cholesterol depletion with mβCD

The PtK2 cells after membrane fusion were treated with 5 mM mβCD for 30 min. The chemical drug-containing medium was replaced with fresh HEPES-buffered medium before SPT.

### Actin cytoskeleton manipulation with CK-666

The actin polymerization was inhibited by treating the cells with 100 μM CK-666. The SPT measurements were performed within 1 h of the treatment in the CK-666-containing cell medium.

### COBRI microscope imaging

A contrast-enhanced coherent brightfield (COBRI) microscopy that was previously demonstrated by our group (47,48) was used for recording the motion of AuNP on the cell surface. The detailed optical setup is plotted in Fig. S1. Briefly, a continuous-wave laser at 532-nm wavelength (Finesse Pure, Laser Quantum) is delivered to the cell sample with a condenser microscope objective (UMPLFLN 20XW, NA0.5, Olympus) at an average illumination intensity of 1 kW/cm^2^. The forward-scattered signal of the AuNP and the non-scattered transmitted reference light were collected by an oil-immersion objective (UPLSAPO 100XO, NA1.4, Olympus) in the transmission geometry. The interference contrast between signal and the reference was enhanced by back-pupil function engineering where a dot-shaped attenuator selectively reduced the amplitude of reference beam by a factor ∼100. The contrast-enhanced COBRI image (digital resolution of 128×128) was recorded by a high-speed complementary metal-oxide-semiconductor (CMOS) camera (v711, Vision Research) at a frame rate of 10,000 fps with an exposure time of 99.638 μs. The overall optical magnification is 471, corresponding to an image pixel size of 48 × 48 nm^2^ on the sample.

### Image postprocessing for background removal

Before localization of the particle by SPT, the nonspecific scattering background of the biological cells was largely removed by temporal median filtering (49,50). A temporal median background was calculated every 2 s. This temporal median background contained features that were relatively stationary, representing the scattering of large cell structures and any non-uniformity of the light illumination. The temporal median background was removed by normalizing the raw images by the corresponding background image. After background removal, the moving particles were clearly observed, whereas the cell was nearly invisible. It was shown that the temporal median filtering effectively suppressed the error of SPT due to the cell background, making it possible to localize the particle with a precision of a few nanometers in the live cellular environments (51).

### SPT

The particle position in the background-corrected image was determined by a least-squares fitting of the local sub-image with a two-dimensional (2D) Gaussian function where the lateral positions, the widths, and the amplitudes of the 2D Gaussian function were kept as free fitting parameters. The lateral localization precision is estimated from the uncertainty of the fitting of the individual particles for each frame (52). The average lateral precision is approximately 6 nm. Connecting the nearest neighboring particle positions in the consecutive frames formed a trajectory. The SPT analysis was performed with the home-written MATLAB codes.

### Calculation of MSD and $D_{app}$ from a diffusion trajectory

We divided the trajectories into segments with 1000 steps. For each 1000-step segment, we calculated the 2D time-average mean squared dispalcement (MSD) as a function of delay time (43,50).

(1) $$MSD\left(n\Delta t\right)=\frac{1}{N-n}\sum_{i=1}^{N-n}{\left\{\vec{r}\left[\left(i+n\right)\Delta t\right]-\vec{r}\left(i\Delta t\right)\right\}}^{2}.$$

where $\vec{r}\left(t\right)$ is the particle position at time *t*, Δt is the frame time, and N is the trajectory length.

The apparent diffusion coefficient $D_{app}$ as a function of time interval was calculated from the MSD as

(2) $$D_{app}\left(n\Delta t\right)=\frac{MSD\left(n\Delta t\right)-MSD\left(\Delta t\right)}{4\left(n-1\right)\Delta t}$$

We emphasize that, in this definition, the first MSD data point at the shortest delay time (i.e., MSD(Δt)) is used as the baseline for the computation of $D_{app}$. Thus, the influence of the localization error, including the static and dynamic errors (53), to the $D_{app}$ is removed by subtraction. This operation provides a reliable detection and characterization of anomalous subdiffusion from the $D_{app}$.

### Multimobility analysis of $D_{app}$

We noted that the distribution of $D_{app}\left(n\Delta t\right)$ measured from the multiple trajectories deviates from a simple normal distribution. To determine the possible composition of the measured $D_{app}$, we analyze it by Gaussian mixture model (GMM) that describes the data with a mixture of multiple Gaussian functions. By examining the residual error and information theoretic criterion (Akaike information criterion and silhouette coefficient) against the number of components, we found that the $D_{app}$ is best described by a dual-mobility mixture model (see the verifications in Fig. S2). The GMM analysis gives the diffusion coefficients of the two mobilities and their uncertainties, corresponding to the centers and widths of the two Gaussian distributions, respectively. The population ratios of the two mobilities are calculated based on the areas of the two Gaussian distributions, respectively. Note that the outliers of $D_{app}$ are excluded from the GMM analysis. The identification of outlier is based on the 1.5× interquartile range (IQR) rule. The GMM analysis was performed with the home-written MATLAB codes.

### Determination of the hop diffusion characteristics

The hop diffusion model considers a Brownian diffuser moving in a 2D square-shaped periodic potential that is semi-permeable. The microscopic diffusion coefficient of the diffuser is $D_{micro}$, and the size of the square potential is L. The resulting MSD can be approximated as the summation of two MSDs of a free diffuser and a confined diffuser with proper weighting (54):

(3) $$MSD\left(\tau \right)=\;\rho \cdot f\left(L^{2},D_{micro},\tau \right)+\left(1-\rho \right)4D_{micro}\tau$$

where

(4) $$f\left(L^{2},D_{micro},\tau \right)=\frac{L^{2}}{3}-\frac{32L^{2}}{\pi^{4}}\sum_{k=1\left(odd\right)}^{\infty }\frac{1}{k^{4}}\text{exp}\left[-{\left(\frac{k\pi }{L}\right)}^{2}D_{micro}\tau \right].$$

ρ is a weighting variable that represents the confinement strength. For example, ρ = 1 when the potential is infinite and thus the particle is locally confined within one compartment. In contrary, ρ = 0 when the potential vanishes and thus the particle diffuses freely. We call ρ as the confinement strength hereinafter. Given the above MSD model in Eq. 3 and the definition of $D_{app}$ in Eq. 2, it is straightforward to write $D_{app}$ as a function of L, $D_{micro}$, and ρ:

(5) $$D_{app}\left(n\Delta t\right)=\rho \frac{8L^{2}}{\pi^{4}\left(n-1\right)\Delta t}\sum_{k=1\left(odd\right)}^{\infty }\frac{1}{k^{4}}e^{-{\left(\frac{k\pi }{L}\right)}^{2}D_{micro}\Delta t}\left(1-e^{-{\left(\frac{k\pi }{L}\right)}^{2}D_{micro}\left(n-1\right)\Delta t}\right)+\left(1-\rho \right)D_{micro}.$$

We fit the experimental data of $D_{app}\left(n\Delta t\right)$ with Eq. 5 where L, $D_{micro}$, and ρ are treated as three free fitting parameters.

### Validation of hop diffusion analysis with simulated trajectories

Hop diffusion trajectories were simulated in periodic diffusion barriers, where the compartment size L and transmission probability (connected to the confinement strength ρ) were freely adjustable parameters. The $D_{micro}$ was set as 0.6 μm^2^/s and the temporal resolution of the simulation was 0.1 ms. Each trajectory started at a random position in the periodic square-shaped compartments. To determine the next position of the particle in the following time point, a random 2D Brownian displacement was generated. Once the particle displacement met the boundary, there was a specified probability for the particle to cross the boundary, or otherwise it was reflected at the boundary. The displacement generation was repeated until the end of the trajectory. We simulated 1000 hop diffusion trajectories (each consists of 1000 steps), and calculated their ensemble $D_{app}$ as a function of delay time ranging from 0.1 ms to 5 ms. The $D_{app}$ was fitted by the analytical approximation of hop diffusion model Eq. 5 from which the compartment size L is estimated.

## Results

### Single phospholipids undergo subdiffusion on the length scale below 100 nm in the cell plasma membrane

**Figure 1:**
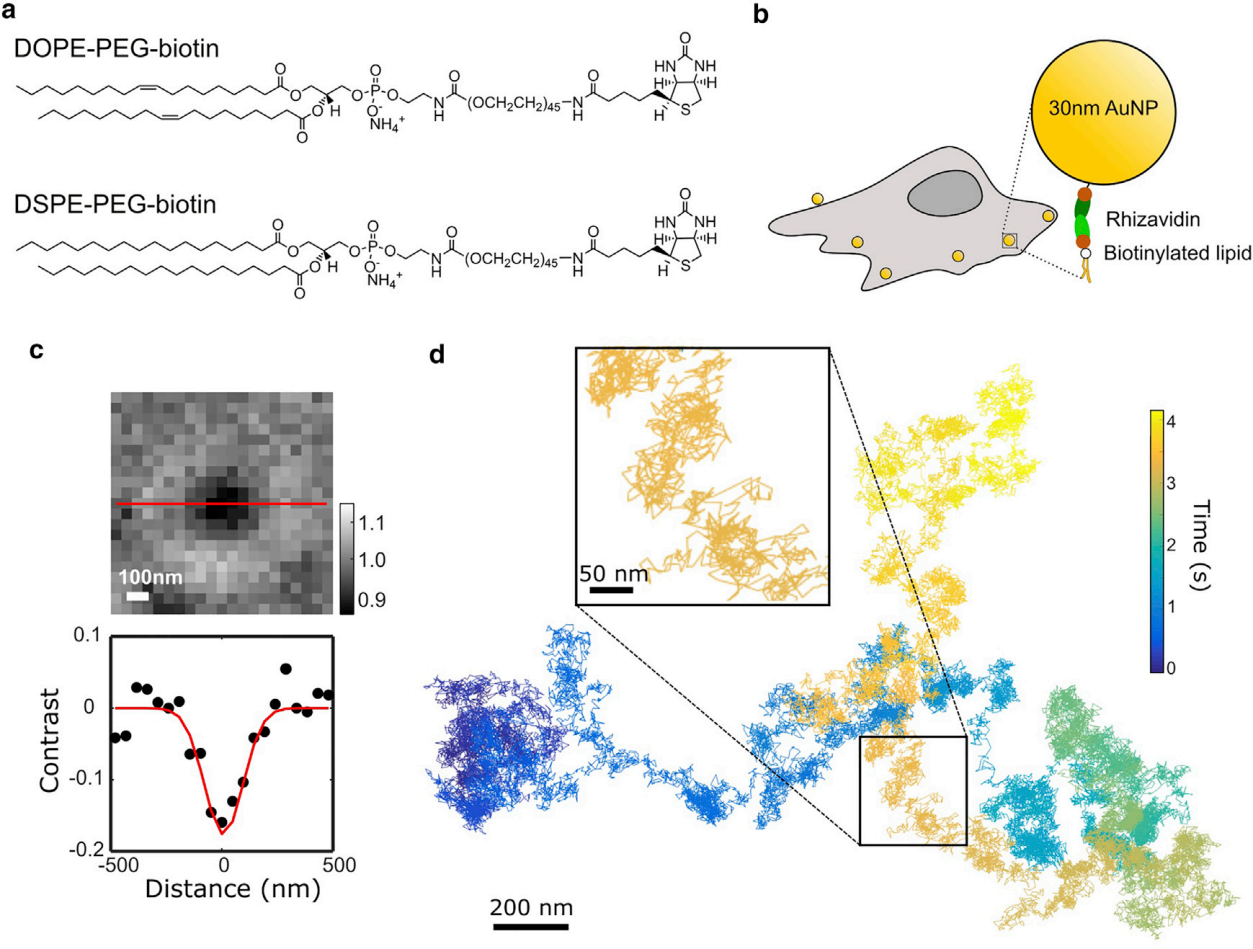
High-speed tracking of single biotinylated phospholipids in the plasma membrane of live cells. (*a*) Structural formula of two synthetic biotinylated lipids that were used in this study. (*b*) Schematic of single-lipid labeling with AuNP on the cell membrane. (*c*) Optical image of AuNP and its intensity line profile. (*d*) A representative diffusion trajectory of a DSPE lipid molecule measured by high-speed molecule tracking at 10,000 fps. To see this figure in color, go online.

We set out to measure the diffusion of single phospholipids in the plasma membrane of live PtK2 cells at 37°C. Two probe lipids are designed for our study (Fig. 1 *a*): the unsaturated phospholipid DOPE-PEG2000-biotin (denoted as DOPE) and the saturated phospholipid DSPE-PEG2000-biotin (denoted as DSPE). The two probe lipids are introduced into the cell plasma membrane separately through membrane fusion (see Materials and methods). The addition of biotinylated probe lipids into the plasma membrane is confirmed by the successful attachment of dye-conjugated streptavidin (Fig. S3). We reason that these two lipids would have different affinities to the putative cholesterol-dependent membrane domains: DOPE prefers staying in the more fluidic Ld phase, while DSPE prefers partitioning in the more ordered Lo/gel phase. The distinct partitioning preference of DOPE and DSPE are observed in the Ld/Lo-phase coexisting model membrane (Fig. S4). Therefore, for the measurements in the cell plasma membrane, we consider DOPE as a non-raft probe lipid, and DSPE as a raft-associated probe lipid (55). By comparing their dynamics, we examine the effects of membrane phases on molecular dynamics.

The two probe lipids are biotinylated in the headgroup for AuNP labeling via biotin-binding proteins (Fig. 1 *b*). We employ the method reported previously of monovalent and oriented AuNP labeling to the biotinylated lipid through a dimeric avidin binding protein, rAv (45). This labeling scheme introduces negligible artifacts to the lipid diffusion, measuring a diffusion coefficient that is comparable with the fluorescence-based methods. The stable scattering signal of the AuNP enables continuous observation at a high speed over a long time. In this work, we capture the motion of single AuNPs by contrast-enhanced COBRI microscopy at a high speed of 10,000 fps (see Materials and methods). The optical contrast of the AuNP is enhanced by back-pupil function engineering, enabling the clear visualization of the AuNP on the cell surface (Fig. 1 *c*). The background scattering of the cell is largely removed by image postprocessing (see Materials and methods). After background removal, the SNR of AuNP on the cell membrane is ∼5, allowing us to determine its 2D spatial position within 6–8 nm (Fig. S5). A continuous diffusion trajectory is reconstructed by connecting the particle position in the consecutive frames. Fig. 1 *d* plots a representative diffusion trajectory of DSPE consisting of more than 40,000 steps (see Video S1). Although the live cell plasma membrane is not flat, containing distinct 3D membrane nanostructures such as clathrin-coated pits, we note that the effects of these structures on our measured lipid diffusion seem not to be significant. By examining our trajectories, we find very rare events of transient trapping of the lipids (covering <1% of the total observation time) that could be due to these local membrane 3D structures. Moreover, we point out that the membrane organization and dynamics of our current investigation are at a small length scale, typically <0.005 μm^2^, in which the long-range height variation of membrane is expected to be negligible (56).

Video S1. A COBRI video of a gold nanoparticle attached to a single lipid diffusing on the live cell membrane Image acquisition rate is 10,000 frames per second. The reconstructed trajectory is plotted.

**Figure 2:**
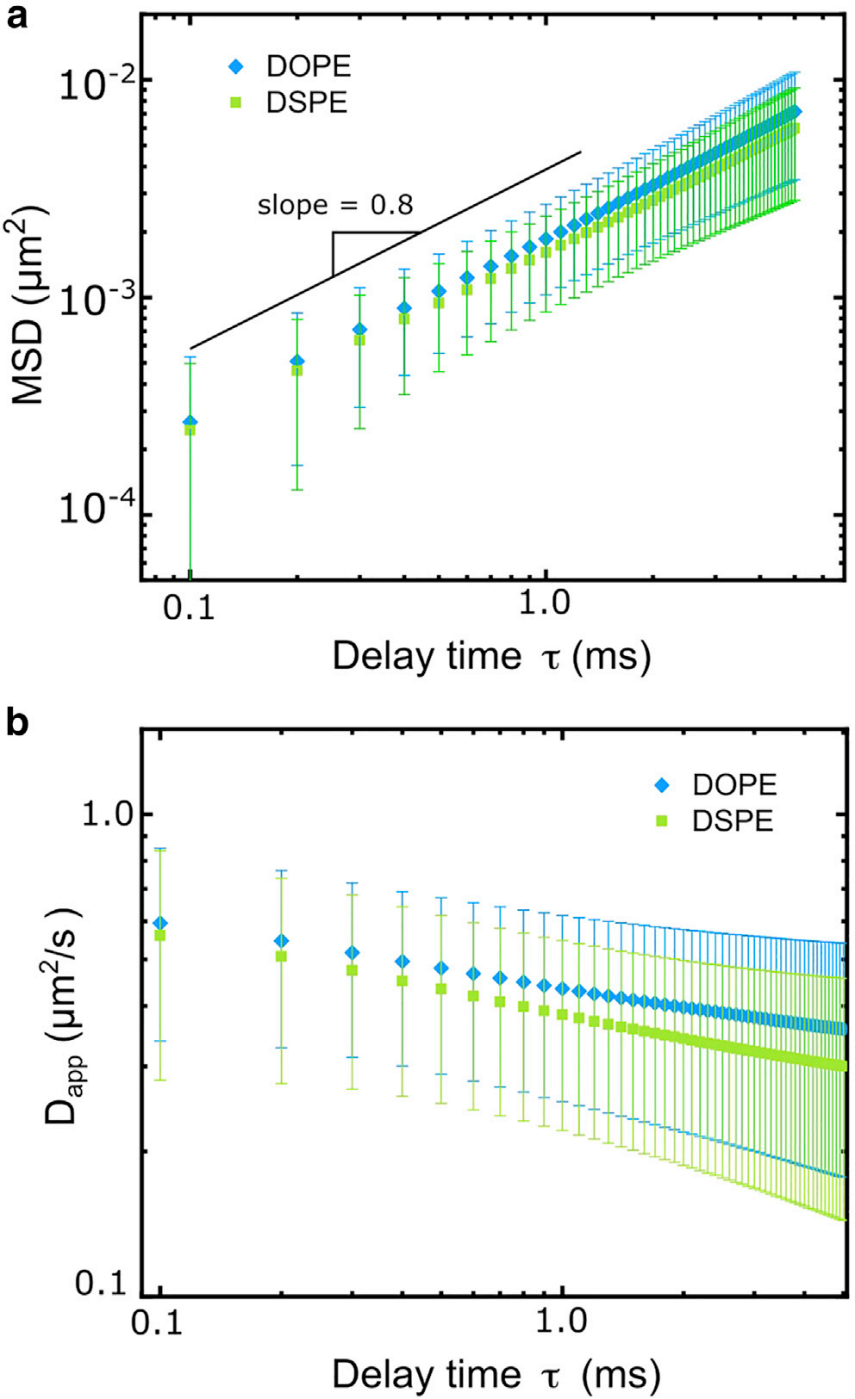
Diffusion characteristics of DOPE and DSPE in the plasma membrane of live PtK2 cells measured at 37°C. (*a*) The ensemble time-average MSDs of DOPE and DSPE in the cell membrane, both showing anomalous subdiffusion. The solid line shows a slope of 0.8. Note that, in the log-log plot of the MSD, the constant offset in the MSD due to the localization error has been removed by subtraction. (*b*) The apparent diffusion coefficient $D_{app}$ of DOPE and DSPE as a function of delay time in the live cell plasma membrane. The decay of $D_{app}$ with the delay time indicates anomalous subdiffusion. The saturated lipid DSPE is more subdiffusive than the unsaturated DOPE in the cell plasma membrane. The error bars represent the standard deviations. To see this figure in color, go online.

We measure around 250 trajectories each for the DOPE and DSPE that are longer than 10,000 steps. The coordinates of these trajectories are available as the supporting material. The ensemble time-averaged MSDs are calculated and displayed in Fig. 2 *a*. The MSD data do not scale linearly with the delay time τ within the range of 0.1–1 ms. For quantitative analysis, the MSD is fitted by the model of anomalous diffusion (43):

(6) $$\text{MSD}\left(\tau \right)=4D_{\alpha }\tau^{\alpha }+\text{C,}$$

where α is the anomalous exponent, $D_{\alpha }$ is the generalized diffusion coefficient, τ=nΔt is the delay time (n is a positive integer and Δt is the frame time), and C is a constant due to the localization error (53). The resulting α of DOPE and DSPE are 0.79 ± 0.16 and 0.77 ± 0.16, respectively, indicating anomalous subdiffusion. To better visualize the nature of subdiffusion, we calculate the apparent diffusion coefficient at different delay times, i.e., $D_{app}\left(\tau =n\Delta t\right)$, based on the MSD data (Eq. 2 in Materials and methods).

For free diffusion, $D_{app}$ is a constant at all timescales. For subdiffusion, $D_{app}$ drops as the timescale increases. We stress that, under our definition of $D_{app}$, the localization error in SPT (including the static and dynamic errors) does not introduce biased detection of the anomalous diffusion (see Materials and methods). The $D_{app}\left(\tau \right)$ of DOPE and DSPE are plotted in Fig. 2 *b*, both showing a decay against the delay time, a signature of subdiffusion. We verify that the measured subdiffusion is not due to the labeling artifacts or the analytical bias because free diffusion is measured in a homogeneous model membrane of supported lipid bilayers (α = 1 and a constant $D_{app}$ shown in Fig. S6). Taken together, both DOPE and DSPE undergo anomalous subdiffusion in the cell plasma membrane in the sub-millisecond timescale. The subdiffusion characteristics of DOPE and DSPE only exhibit small discrepancies in the cells of normal conditions, but these differences are statistically significant in our high-speed SPT data. Our data show that DOPE diffuses more freely than DSPE in the plasma membrane on the length scale below 100 nm, which leads to a faster diffusion on the macroscopic length scale.

### Spatiotemporal heterogeneous diffusion of phospholipids in the cell plasma membrane

**Figure 3:**
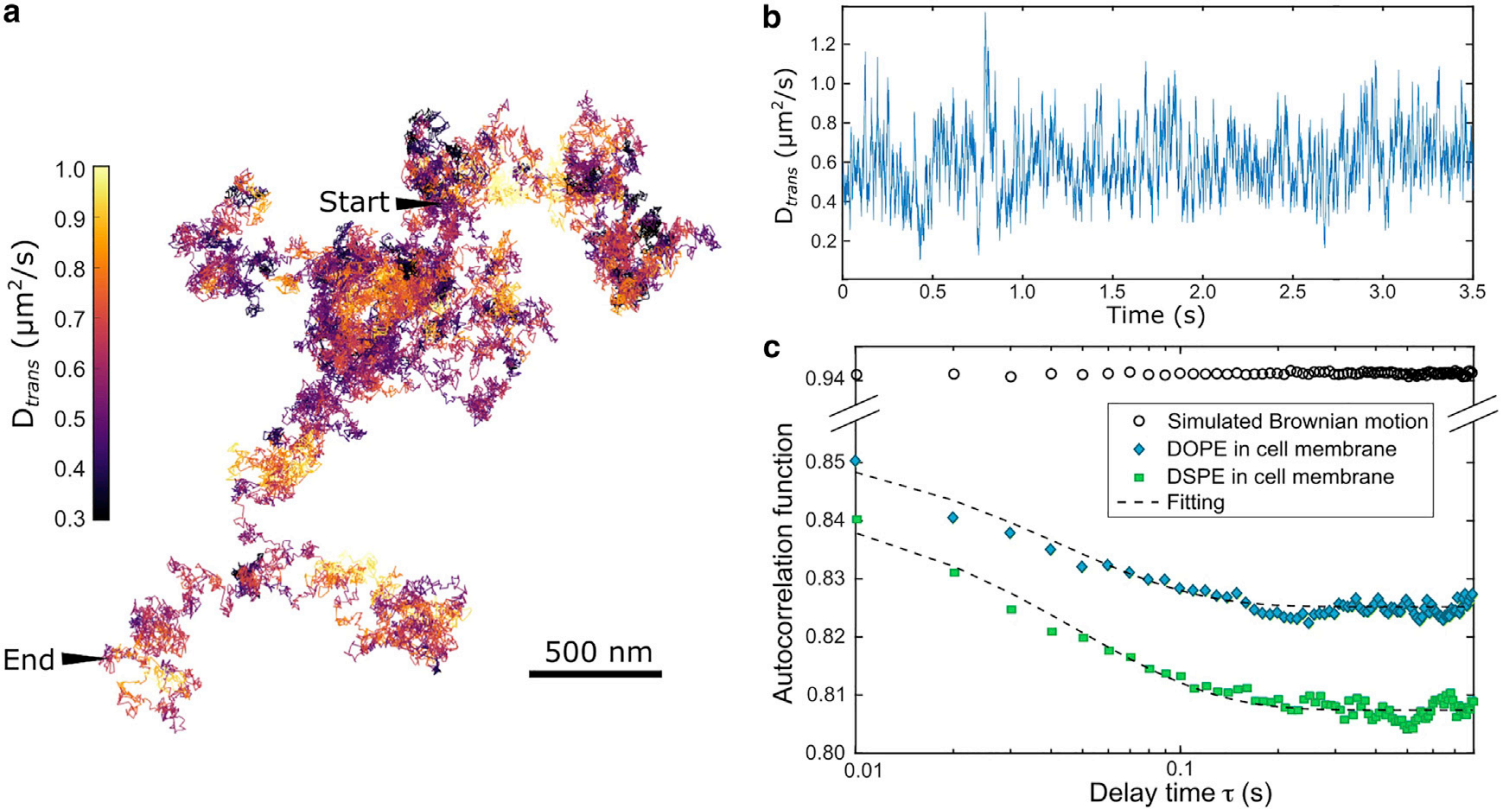
Large variation in the transient diffusion coefficient ($D_{trans}$) of single phospholipids in the cell membrane. (*a*) Diffusion trajectory of DOPE color-coded with its $D_{trans}$. (*b*) Time trace of the $D_{trans}$ of the trajectory plotted in (*a*). (*c*) Autocorrelation functions (ACFs) of $D_{trans}$ of DOPE and DSPE in the cell membrane with the corresponding exponential fittings. The ACF of simulated Brownian motion is plotted for compassion. To see this figure in color, go online.

To explore the possible reasons for the anomalous subdiffusion, we examine the transient diffusion behaviors by calculating the transient diffusion coefficient ($D_{trans}$) for every trajectory segment of 100 steps (corresponding to a time window of 10 ms). Here, the $D_{trans}$ is defined as the apparent diffusion coefficient at the shortest time interval; i.e., $D_{app}\left(\Delta t=0.1\text{ms}\right)$ (see Materials and methods). The result of $D_{trans}$ of a representative trajectory of DOPE is plotted in Fig. 3 *a* (see Video S2). In this trajectory, the $D_{trans}$ varies considerably in time, ranging from 0.2 μm^2^/s to 1.2 μm^2^/s, approximately (peak to peak; see Fig. 3 *b*). We note that such variation is greater than the stochastic fluctuation of Brownian motion. We confirm that the large $D_{trans}$ variation appears in all trajectories measured in the cell membrane. Specifically, the spreads of $D_{trans}$, defined as the range between the 25^th^ percentile and the 75^th^ percentile, are 0.36–0.78 μm^2^/s and 0.31–0.70 μm^2^/s for DOPE and DSPE, respectively (Table 1), whereas the Brownian motion of 0.6 μm^2^/s has a spread of $D_{trans}$ of 0.50–0.69 μm^2^/s.

**Table 1:** The hop diffusion characteristics of the two mobilities of DOPE and DSPE in the plasma membrane of live PtK2 cells

			Spread of $D_{trans}$ (μm^2^/s)	$D_{trans}$ correlation time (ms)	Mobility	Compartment size L (nm)	Confinement strength (ρ)	Microscopic diffusion coefficient $D_{micro}$ (μm^2^/s)
37°C	DOPE	0.36–0.78		53 ± 5	fast	79 ± 4	0.50 ± 0.01	0.84 ± 0.02
					slow	67 ± 2	0.57 ± 0.01	0.49 ± 0.01
	DSPE	0.31–0.70		69 ± 5	fast	62 ± 1	0.56 ± 0.01	0.86 ± 0.02
					slow	51 ± 1	0.61 ± 0.01	0.47 ± 0.01
37°C +mβCD	DOPE	0.26–0.59		47 ± 5	fast	46 ± 1	0.64 ± 0.01	0.79 ± 0.03
					slow	40 ± 1	0.68 ± 0.01	0.45 ± 0.01
	DSPE	0.24–0.53		43 ± 5	fast	44 ± 1	0.78 ± 0.01	1.23 ± 0.07
					slow	40 ± 1	0.73 ± 0.01	0.52 ± 0.02
25°C	DOPE	0.28–0.61		47 ± 6	fast	53 ± 2	0.55 ± 0.01	0.70 ± 0.02
					slow	56 ± 1	0.59 ± 0.01	0.38 ± 0.01
	DSPE	0.26–0.63		50 ± 5	fast	47 ± 1	0.63 ± 0.01	0.82 ± 0.01
					slow	40 ± 1	0.61 ± 0.01	0.42 ± 0.01
25°C +mβCD	DOPE	0.21–0.56		47 ± 5	fast	41 ± 1	0.79 ± 0.01	1.03 ± 0.05
					slow	39 ± 1	0.73 ± 0.01	0.40 ± 0.01
	DSPE	0.17–0.47		44 ± 6	fast	38 ± 1	0.81 ± 0.02	1.12 ± 0.13
					slow	35 ± 1	0.70 ± 0.01	0.37 ± 0.01

Video S2. A diffusion trajectory of DOPE in the cell membrane color coded with the transient diffusion coefficient

The wide variation in $D_{trans}$ indicates that the diffusion in the cell membrane is heterogeneous in space and in time. There are at least two possible reasons for the variation in $D_{trans}$. First, the membrane organization could be spatially heterogeneous, which leads to the spatially varying diffusion characteristics of the lipids. The other possible reason is the dynamic association of the lipid to different molecular complexes. Once the lipid gets associated with a molecular cluster, the measured diffusion is determined by the movement of the whole molecular complex. Thus, dynamic association with different complexes results in a variation of measured diffusion coefficient. More discussion about molecular complex is presented in the Discussion section. These two mechanisms are not exclusive, and they could affect the lipid diffusion simultaneously. Although we cannot distinguish explicitly these two effects with the current design of our experiments, we note that very different values of $D_{trans}$ are measured in the same local area within a short time delay. Thus, the role of dynamic molecular association should not be neglected.

We further analyze the fluctuation statistics of $D_{trans}$ by calculating its temporal autocorrelation function (ACF). Both DOPE and DSPE show decays in their ACFs of $D_{trans}$ (Fig. 3 *c*). In contrast, the simulated Brownian motion exhibits a highly correlated $D_{trans}$ and thus a constant in the ACF. The decay in ACF indicates that the diffusion changes over time, most likely due to the heterogeneous membrane organization and dynamic association to membrane complexes. By fitting the ACF with an exponential decay, we find that the correlation time of $D_{trans}$ for DOPE is 53 ± 5 ms, which is shorter than that of DSPE (69 ± 5 ms). The shorter correlation time of DOPE may be due to its tendency to partition in the more disordered membrane domains, leading to fast-changing nanoscopic diffusion characteristics. On the other hand, the saturated lipid DSPE exhibits a longer correlation time, which suggests its association to the more ordered domains that are relatively more stable and slowly varying.

### Phospholipids exhibit two-mobility anomalous hop diffusion in the sub-millisecond timescales

**Figure 4:**
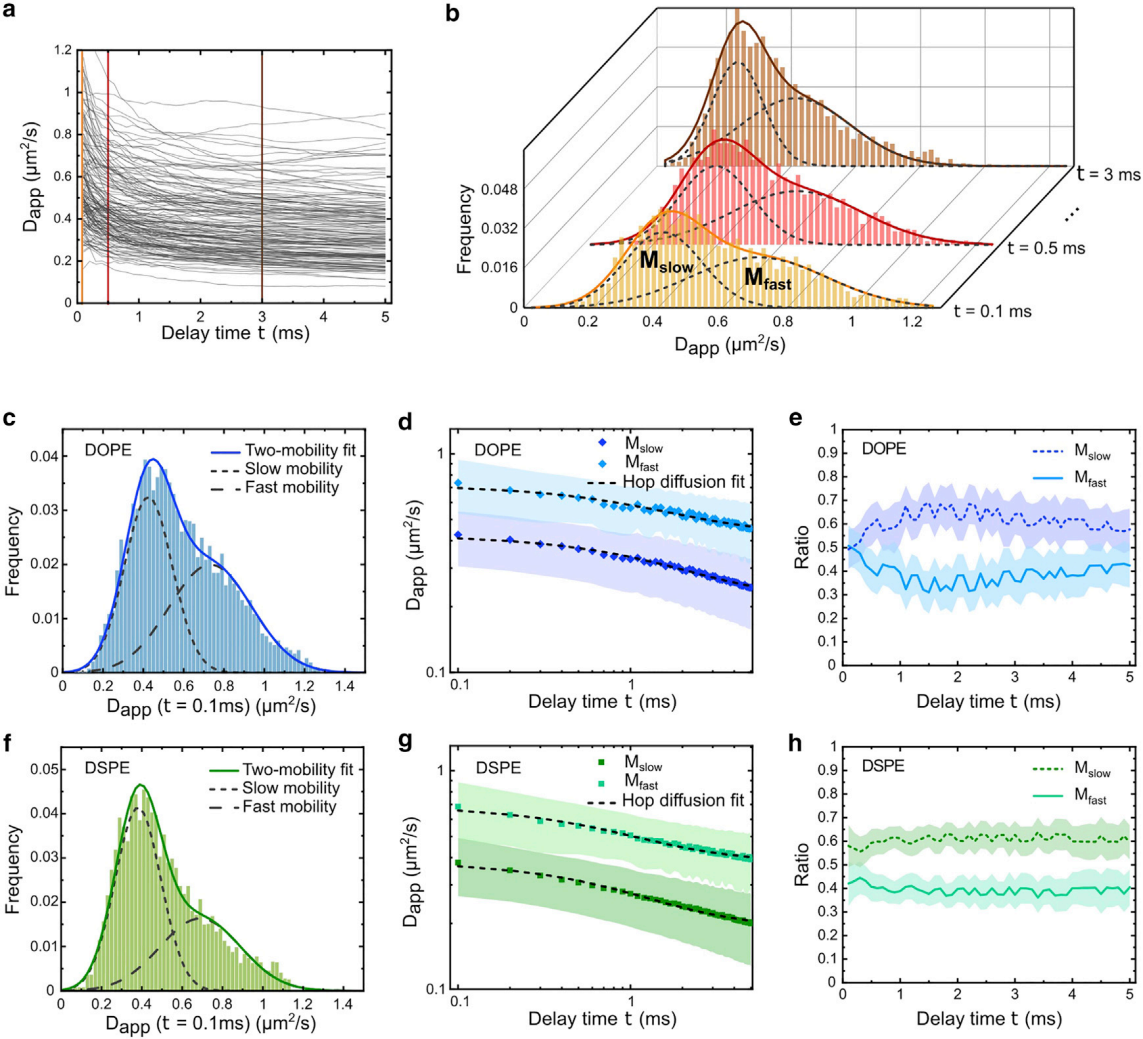
Dual-mobility subdiffusion of DOPE and DSPE in the cell plasma membrane measured at 37°C. (*a*) $D_{app}$ as a function of delay time of the individual trajectory segments of DOPE. (*b*) Representative histograms of $D_{app}$ of DOPE at τ = 0.1, 0.5, and 3 ms. The histograms are well fitted by the superpositions of two Gaussian functions, indicating that the lipid diffuses with two mobilities in the membrane, denoted as M_fast_ and M_slow_. (*c*–*e*) Dual-mobility results of DOPE. (*f*–*h*) Dual-mobility results of DSPE. (*c* and *f*) Histograms of $D_{app}$ at τ = 0.1 ms for DOPE (*c*) and DSPE (*f*). (*d* and *g*) $D_{app}\left(\tau \right)$ of M_fast_ and M_slow_ for DOPE (*d*) and DSPE (*g*). The dashed curves are the corresponding fittings with the hop diffusion model. (*e* and *h*) Population ratios of the two mobilities for DOPE (*e*) and DSPE (*h*) as a function of delay time. The M_fast_ and M_slow_ are ∼40% and 60% of the total population, respectively. The shaded areas plotted in (*d* and *g*) and (*e* and *h*) represent the widths of the distributions and the accuracies of the fittings, respectively. To see this figure in color, go online.

We examine quantitatively the distributions of the transient diffusion characteristics at different time intervals. The trajectories are segmented into short ones with an equal length of 1000 steps (the remaining trajectory segments shorter than 1000 steps are discarded). For each segment, we compute the $D_{app}$ as a function of delay time (se Materials and methods). The results of DOPE are displayed in Fig. 4 *a*. We examine the distribution of $D_{app}$ at every delay time (e.g., the histograms of $D_{app}$ at τ = 0.1, 0.5, and 3 ms are plotted in Fig. 4 *b*). The large amount of data allows us to analyze the distribution of $D_{app}$ with high accuracy. While there is clearly a peak in the histogram of $D_{app}$, we note that the histogram deviates from a single normal distribution. We examine the multimobility nature of $D_{app}$ and find that the histograms of $D_{app}$ can be well described by a superpositions of two distinct mobilities (see Materials and methods and Fig. S2 for the multimobility analysis). In the case of DOPE at τ= 0.1 ms, the two mobilities are 0.74 ± 0.20 μm^2^/s and 0.43 ± 0.12 μm^2^/s with the population fractions of 49% and 51%, respectively (Fig. 4 *c*). Meanwhile, for DSPE, the two mobilities (fractions) are 0.68 ± 0.20 μm^2^/s (42%) and 0.38 ± 0.11 μm^2^/s (58%), respectively (Fig. 4 *f*). The comparisons of these diffusion coefficients with the previously reported values are presented in the Discussion section. We call these two mobilities the fast mobility (M_fast_) and the slow mobility (M_slow_) hereinafter. The dual-mobility fitting of $D_{app}$ is performed at every time delay ranging from 100 μs to 5 ms, giving the $D_{app}\left(\tau \right)$ of the two mobilities (Fig. 4 *d* and *g* for DOPE and DSPE, respectively). We note that both mobilities exhibit decays as the delay time increases, and thus they are both subdiffusive. Furthermore, the population fractions of the two mobilities stay relatively unchanged over the timescales of our measurement from 0.1 to 5 ms (∼40% for M_fast_ and ∼60% for M_slow_, shown in Figs. 4 *e* and 3 *h*). The co-existence of the two mobilities agrees with our observations that the lipids undergo spatiotemporally heterogeneous diffusion in the cell membrane at the nanoscale. As a control, we measure a normal diffusion of DOPE in the model membrane of supported lipid bilayers (Fig. S6).

In the dual-mobility data, DOPE has a slightly higher mobility than DSPE in all the timescales. Moreover, the $D_{app}$ of DOPE drops more slowly than DSPE, meaning that the diffusion of DOPE is less subdiffusive. Previous studies have shown that the subdiffusion of phospholipid in the sub-millisecond timescale is closely connected to the membrane compartmentalization by the cell cytoskeleton (18,57). According to the pickets-and-fences model (14), the movement of phospholipids located in the outer leaflet of the plasma membrane is hindered by the transmembrane proteins that are anchored to and aligned along the actin-based membrane skeleton meshwork underneath the membrane.

To analyze the subdiffusion of the two mobilities, we adopt a hop diffusion model that is often used to describe the diffusion in compartmentalized membrane by cytoskeleton (54). This hop diffusion model represents the motion of a free Brownian diffuser with a diffusion coefficient of $D_{micro}$ in a periodic square-shaped semi-permeable barrier with a dimension of L and a confinement strength of ρ (see Materials and methods for the details). Although the hop diffusion model does not account for many aspects of the real molecular interactions and membrane organizations at the nanoscale, it is considered a reasonable approximation by giving comprehensive outputs of the effective compartment size and confinement strength, which are useful for characterizing the membrane compartmentalization. The validity of the hop diffusion analysis is examined based on the simulated data (see Materials and methods and Fig. S7). We note that the compartment size L is estimated accurately by the hop diffusion analysis except for the weakly confined cases (the transmission probability greater than 0.01). This is because, in the weakly confined scenarios, the particle tends not to explore the whole compartment area before hopping to the adjacent zone, leading to an underestimated compartment size.

We fit our experimental data of $D_{app}$ of the two mobilities separately with the hop diffusion model. Each fitting contains $D_{micro}$, L, and ρ as the three free parameters (see Materials and methods). The hop diffusion model describes the two mobilities of DOPE and DSPE very well (Fig. 4 *d* and *g*). The fitting results of the two mobilities of DOPE and DSPE are summarized in Table 1. The effectiveness of hop diffusion in describing our subdiffusion data is further supported by the good agreement between the simulated hop diffusion data and the experimental results (Fig. S8; Table S1). We point out that the subdiffusion is observed in the simulated hop diffusion at the shortest timescale of 0.1 ms even when a Brownian diffuser is considered in the hop diffusion model (Fig. S8). It means that the compartmentalization affects the measured diffusion mode at the shortest timescale of our experiments. A higher temporal resolution is needed to delineate the true lipid diffusion mode within the compartment. With the temporal resolution of the current study, we consider the lipid diffusion within the compartment is apparently free.

Our analyses show that the microscopic diffusion coefficients $D_{micro}$ of DOPE are very close to those of DSPE (0.84 (0.49) μm^2^/s for the M_fast_ (M_slow_) of DOPE versus 0.86 (0.47) μm^2^/s for the M_fast_ (M_slow_) of DSPE). Meanwhile, the effective compartment sizes L of DOPE are greater than those of DSPE (79 (67) nm for the M_fast_ (M_slow_) of DOPE versus 62 (51) nm for the M_fast_ (M_slow_) of DSPE). These sizes are comparable with the cytoskeleton mesh size reported previously (13,18). Furthermore, our data show that the confinement strength ρ of DOPE is slightly weaker than that of DSPE (0.50 (0.57) for M_fast_ (M_slow_) of DOPE versus 0.56 (0.61) for M_fast_ (M_slow_) of DPSE). The different confinement properties experienced by DOPE and DSPE (in size and in strength) could be due to the different molecular clusters with which the lipids are associated (1,3,58). For example, DSPE is expected to partition into and diffuse together with the more ordered membrane nanodomains. In contrast, DOPE prefers moving with molecules in the disordered fluidic phases. The different size and hydrodynamic properties of the clusters would result in a different effective compartment size and strength. The relevance of our SPT data to the hop diffusion model is further supported by the measurement where the cortical actin meshwork is manipulated by chemical drug treatment. By treating the cells with CK-666, which inhibits the actin polymerization (see Materials and methods), we measure an increase in the compartment size L (Fig. S9) as the result of a larger size of the cortical actin meshwork (18).

In sum, we measured a more confined diffusion for the saturated lipid DSPE than the unsaturated lipid DOPE. The confined diffusion can be described by a minimal model of hop diffusion that is sufficient for describing the reduction of apparent diffusion coefficient over the spatiotemporal scales of our investigation. The confinement properties are estimated quantitatively with the effective compartment size L and the confinement strength ρ. Our data show that the saturated lipid DSPE experiences a denser cytoskeletal barrier together with a stronger confinement strength than the unsaturated lipid DOPE.

### Cholesterol modulates the nanoscale subdiffusion of phospholipids in cell plasma membranes

We examine the dependency of nanoscale lipid diffusion on the cholesterol concentration by depleting the cholesterol in the membrane. Previous fluorescence-based studies observe a slower lipid diffusion in cell plasma membrane after cholesterol depletion (measured in a much larger spatiotemporal regime) (35). It was proposed that the reduction of cholesterol concentration induces solid-like membrane domains in gel phase, and these gel-phase nanodomains are thought to act as diffusion obstacles for the membrane molecules. The above description is particularly true for unsaturated lipids (e.g., DOPE) that prefer the disordered phase. However, for the saturated lipids (e.g., DSPE) that prefer the ordered phases, it could readily partition in the highly ordered, gel-like membrane nanodomains, especially when the Lo membrane fraction is reduced by cholesterol depletion. Therefore, because of the distinct preferences to the specific membrane phases of DOPE and DSPE, their diffusion characteristics are expected to be modulated differently by cholesterol depletion.

We measure the nanoscopic diffusion of DOPE and DSPE in the cell plasma membrane after cholesterol depletion. The cells are treated with the chemical drug mβCD at 5 mM for 30 min, after which 30%–40% of cholesterol is expected to be removed from the plasma membrane (59). SPT is conducted for DOPE and DSPE immediately after the treatment. We first calculate the $D_{trans}$ and its fluctuation statistics. The spreads of $D_{trans}$ for DOPE and DSPE in the mβCD-treated cells are 0.26–0.59 μm^2^/s and 0.24–0.53 μm^2^/s, respectively (Table 1). These spreads remain significant when compared with the Brownian motion. Importantly, we note that the correlation times of $D_{trans}$ for DOPE and DSPE become shorter after cholesterol depletion (47 ± 5 ms and 43 ± 5 ms for DOPE and DSPE, respectively). The shorter correlation times suggest that the membrane organization is more heterogeneous. This could be because the mβCD treatment creates the solid-like nanodomains that act as diffusion obstacles of DOPE and DSPE, making their diffusion behaviors fluctuate more rapidly.

**Figure 5:**
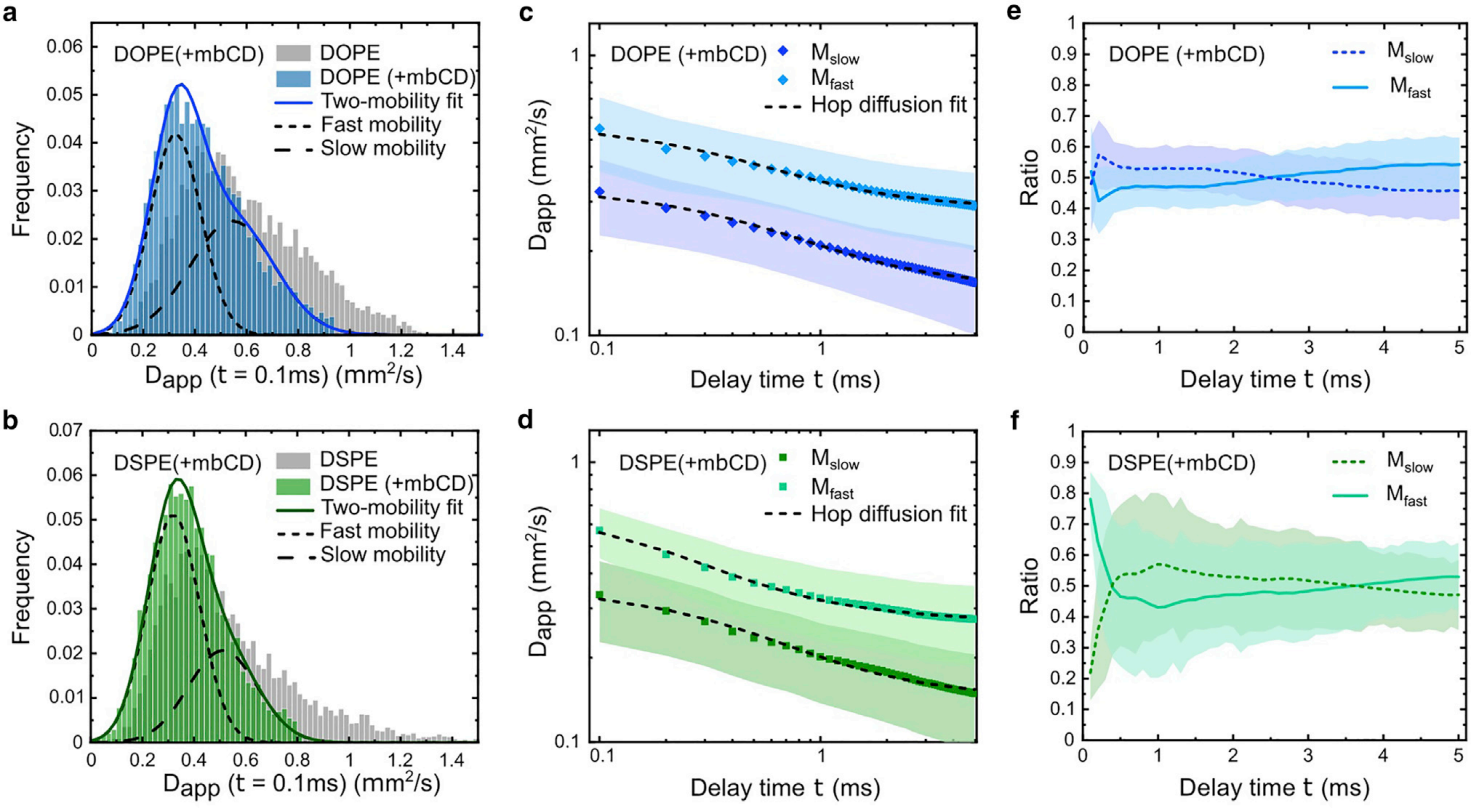
Dual-mobility anomalous subdiffusion of DOPE and DSPE measured in the cholesterol-depleted cell plasma membrane. (*a* and *b*) Histograms of $D_{app}$ at the time delay of 0.1 ms of DOPE (*a*) and DSPE (*b*). The two dashed curves correspond to the two Gaussian functions for the fitting. The data measured in normal cells (without cholesterol depletion) are plotted in gray for comparison. For both DOPE and DSPE, $D_{app}$ is reduced by cholesterol depletion. (*c* and *d*) $D_{app}$ as a function of delay time of the two mobilities (M_fast_ and M_slow_) of DOPE (*c*) and DSPE (*d*). The dashed line shows the fitting with the hop diffusion model. (*e* and *f*) Population ratios of the two mobilities as a function of delay time of DOPE (*e*) and DSPE (*f*). The ratios are mostly 50% ± 10% for the M_fast_ and M_slow_ of the two probe lipids. The shaded areas plotted in (*c*)–(*f*) represent the widths of the distributions and the accuracies of the fittings, respectively. To see this figure in color, go online.

We then examine the distributions of $D_{app}$ at the shortest time interval of 100 μs for the two lipids (Fig. 5 *a* and *b*). In both cases, a reduction in $D_{app}$ is measured after cholesterol depletion. A slower molecular diffusion in the cholesterol-depleted cell membrane was measured before in a much greater spatiotemporal scale (35,36). To elucidate the reasons for the slower diffusion, we perform the analytical methods aforementioned and resolve the dual-mobility hop diffusion for both lipids (Fig. 5 *c*–*f*; Table 1). Our data show that, for both mobilities (M_fast_ and M_slow_) of DOPE and DSPE, the diffusion becomes more constrained with a smaller compartment size (smaller L) and a stronger confinement strength (larger ρ) in the mβCD-treated cells. The reduction of L together with the increase of ρ is unlikely due to the change of cytoskeleton meshwork. Besides, we verify that the membrane topography below ∼100 nm remains statistically unchanged after cholesterol depletion (estimated from the particle contrast; see Fig. S10). Thus, we exclude the possibility that the different diffusion characteristics measured in the mβCD-treated cells are biased interpretation by projecting a 3D diffusion trajectory into 2D.

We attribute the dependency of subdiffusion on cholesterol concentration to the remodeling of phase-separated membrane nanodomains. The two probe lipids of DOPE and DSPE have distinct preferences to the membrane phases, so there could be differences in their cholesterol-dependent subdiffusion behaviors. In our data of DOPE, after cholesterol depletion, the compartment size L drops from 79 (67) nm to 46 (40) nm for M_fast_ (M_slow_). Meanwhile, the confinement strength ρ increases from 0.50 (0.57) to 0.64 (0.68) for M_fast_ (M_slow_) of DOPE. Furthermore, we find that the $D_{micro}$ of DOPE is relatively unchanged by cholesterol depletion, indicating that the nanoscopic fluidity of the disordered phase is not altered significantly by cholesterol depletion. The more confined diffusion of DOPE measured by our high-speed SPT suggests that DOPE undergoes more obstructed diffusion within the fluidic phase between the gel-phase nanodomains.

It is informative to compare the subdiffusion of DOPE and DSPE after cholesterol depletion. Unlike DOPE, DSPE prefers the more ordered phases, and thus it may associate with the newly formed gel-phase nanodomains or the residual Lo-phase membrane fraction. The data show that DSPE experiences a stronger confinement possibly because it is more difficult for the ordered-phase nanodomain to get around the gel-phase obstacles and the immobilized diffusion barriers (including those created by cytoskeleton-binding transmembrane proteins) (60,61). Quantitatively, for DSPE, the compartment size L decreases from 62 (51) nm to 44 (40) nm for M_fast_ (M_slow_). The confinement strength ρ increases from 0.56 (0.61) to 0.78 (0.73) for M_fast_ (M_slow_). Furthermore, unlike DOPE, which has a similar $D_{micro}$ before and after the mβCD treatment, the $D_{micro}$ of DSPE is increased considerably by cholesterol depletion (∼30% (10%) increase for the M_fast_ (M_slow_)). The increase in $D_{micro}$ indicates a smaller domain size, suggesting that the cholesterol depletion creates small membrane domains of ordered phases. The DSPE partitions into these ordered membrane nanodomains and experiences a highly restricted diffusion after cholesterol depletion. Finally, a slight but noticeable change in the population ratio of M_fast_ and M_slow_ is observed due to the cholesterol depletion. The ratio of M_fast_:M_slow_ is approximately 40:60 in the untreated cells (Fig. 4 *e* and *h*) and it becomes roughly 50:50 in the cholesterol-depleted cells (Fig. 5 *e* and *f*). This observation suggests that the lipid diffusion becomes more heterogeneous after cholesterol depletion.

### Nanoscopic diffusion of phospholipids becomes more restricted at a lower temperature

We further examine the effect of cholesterol-mediated phase separation on the membrane dynamics by changing the temperature. Specifically, the SPT measurements are performed on the live cell plasma membrane at 25°C. From a thermodynamics point of view, a slower diffusion is expected at a lower temperature due to the reduced thermal fluctuation. Importantly, local phase separation of the cell membrane may exhibit a strong dependency on the temperature. For example, pronounced phase separations were observed in the giant plasma membrane vesicles (GPMVs) below the physiological temperature (62,63).

**Figure 6:**
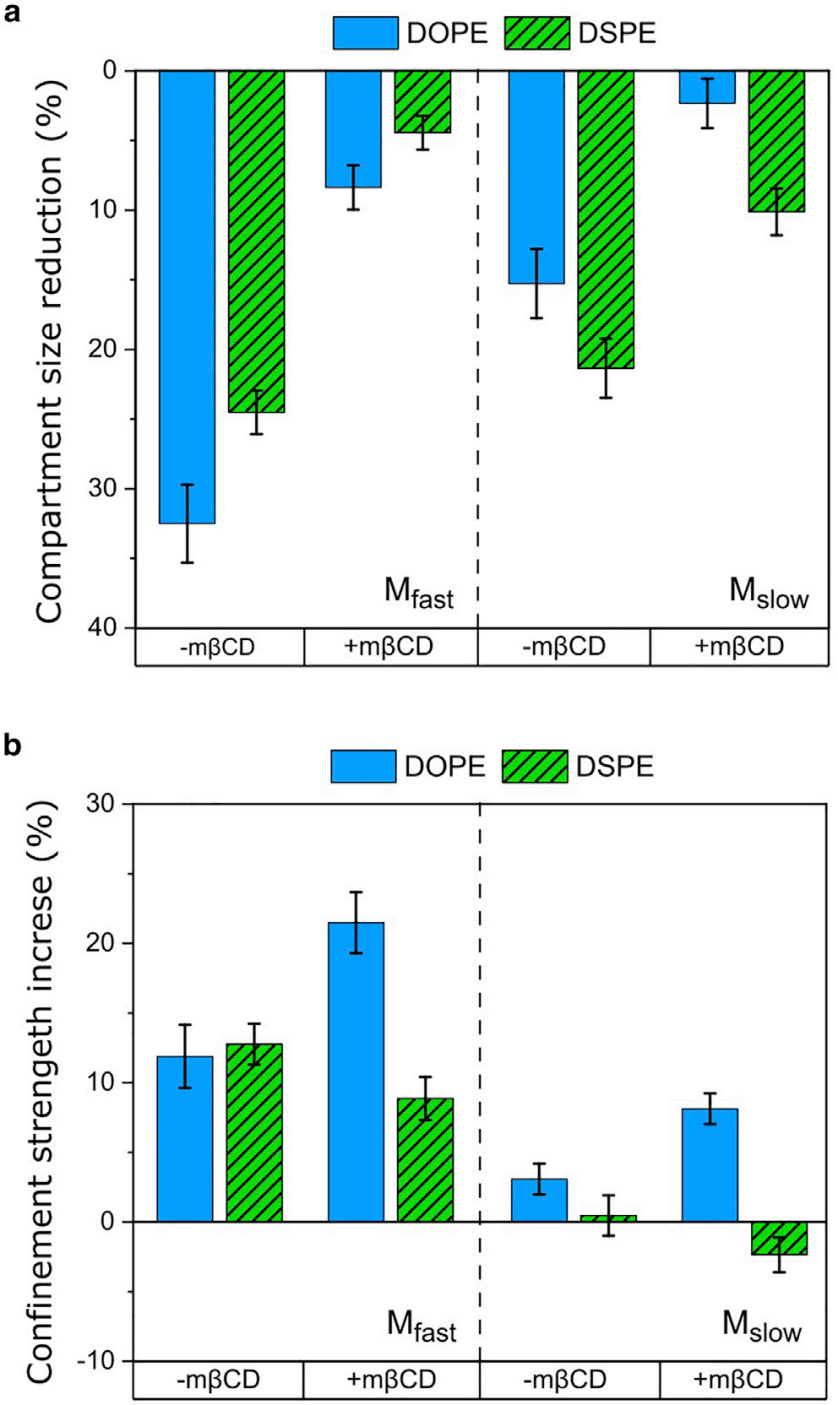
(*a* and *b*) Reduction of the compartment size L (*a*) and increase of the confinement strength ρ (*b*) of the two mobilities (M_fast_ and M_slow_) of DOPE and DSPE by lowering the temperature from 37°C to 25°C. The temperature reduction results in the apparently smaller compartment sizes with the enhanced confinement strengths. The error bars represent the estimation uncertainties. To see this figure in color, go online.

We measured a significantly slower diffusion for DOPE and DSPE at the low temperature (Table 1). The $D_{trans}$ of the two lipids exhibits a shorter correlation time at 25°C, indicating the more heterogeneous diffusion. Moreover, our dual-mobility analyses show that both DOPE and DSPE undergo more restricted diffusion at the lower temperature (smaller compartment size L and a greater confinement strength ρ; data summarized in Fig. 6 and Table 1). These effects of temperature reduction on nanoscopic lipid diffusion are similar to those of cholesterol depletion, suggesting that both treatments affect the lipid diffusion through a change of membrane phase transition. We confirm that the depletion of cholesterol at 25°C further reduces the mobility of the two lipids and makes the diffusion highly restricted (Fig. 6; Table 1).

## Discussion

### Anomalous subdiffusion of phospholipids in the compartmentalized cell plasma membranes below 100 nm

Our high-speed SPT trajectory data reveal the non-Brownian subdiffusion of single phospholipids in the plasma membrane below 100 nm. The unsaturated and saturated lipids (DOPE and DSPE) exhibit similar anomalous exponents (just under 0.8), and both show the characteristic decrease in $D_{app}$ as the delay time is increased. The lipids have similar $D_{app}$ at the shortest timescale of 0.1 ms, but at the longer timescale of 5 ms there is significant difference, with DOPE diffusing faster than DSPE. Anomalous subdiffusion of phospholipids over a similar spatiotemporal scale was measured previously by Kusumi’s group with high-speed SPT (14,57). Compared with Kusumi’s results, our data show a slower $D_{micro}$ (∼0.8 μm^2^/s of our data versus ∼5 μm^2^/s of Kusumi’s data). Meanwhile, we note that the $D_{micro}$ of our measurements agree well with those of the STED-FCS experiments (18,41). It has been proposed that the previously reported $D_{micro}$ by high-speed SPT could be overestimated due to the measurement errors (64). In this work, using the monovalent labeling of 30 nm AuNP and unbiased data analysis, we revisited the high-speed SPT experiments and measured a $D_{micro}$ that agrees with the fluorescence-based STED-FCS results.

In addition, with a large amount of trajectory data, we are able to examine the nanoscale lipid diffusion with unprecedented clarity. We clearly resolve the dual-mobility lipid dynamics that have not been detected in the previous studies. To compare our results directly with Kusumi’s, we use the hop diffusion model established previously to describe the subdiffusion of the two mobilities of our data. We found a larger compartment size for the two mobilities compared with the previous results (∼80 nm and ∼60 nm for the M_fast_ and M_slow_ of DOPE, respectively, whereas Kusumi reported a 43-nm compartment size for the PtK2 cell). In addition, we measure a more confined diffusion for the saturated lipid than the unsaturated lipid. Cholesterol depletion and temperature reduction further make the diffusion more restricted. All these observations indicate that the lipid hop diffusion is governed not only by the cytoskeleton meshwork but also by the membrane phase separation and molecular partitioning.

### Analytical methods for detecting spatiotemporally heterogeneous dual-mobility diffusion

Previous studies have reported heterogeneous and multimobility diffusion of membrane molecules in the cell plasma membrane. By analyzing diffusion trajectories of single-cell plasma membrane molecules, subpopulations were detected for ion channels in cultured neurons (65) and the short transmembrane proteins in the T cells (66). Characterization of heterogeneous diffusion with subpopulations is difficult due to the stochastic nature of diffusion. Reliable detection of a diffusion mode requires consistent measurements of the diffusive motion that lasts for a sufficient number of observations. This criterion precludes the detection of highly transient diffusion modes and fast switching between multiple modes because they are averaged out during statistical analysis. To address this challenge, in this work, we demonstrated an analytical approach to resolve the coexisting multiple mobilities from the SPT trajectory data. Instead of determining the time-dependent diffusion mode directly from the trajectory segments (which requires a large number of steps to ensure statistical accuracy and thus lowers the time resolution), we calculate the MSDs of all trajectory segments and detect the subpopulations from the distributions of the MSD data. Our method is conceptually similar to the multi-component analysis of the cumulative distribution function (CDF) of the particle displacement (50,67). The advantage of analyzing the MSD data over the CDF data is to avoid the complications caused by the localization error. For the high-speed diffusion measurements of membrane molecules, the average displacement in the sub-millisecond timescale is on the order of 1–10 nm, which is comparable with the localization error. As a result, resolving the multimobility through the CDF analysis becomes difficult because the true displacement and the localization error are indistinguishable in the CDF data. In contrast, in the MSD data, the localization error produces a constant offset that allows for straightforward removal from the multimobility analysis. Using our analytical method, the dual-mobility subdiffusion was reliably resolved in the high-speed SPT data.

### Single phospholipids may diffuse with membrane molecular clusters

In the PtK2 cell plasma membrane at 37°C, the microscopic diffusion coefficients $D_{micro}$ of DOPE and DSPE are comparable, both showing 0.85 ± 0.02 μm^2^/s and 0.50 ± 0.03 μm^2^/s for the fast and slow mobilities, respectively (see Table 1). The $D_{micro}$ represents the diffusion coefficient on the length scale of 10–20 nm ($\sqrt{4D_{micro}\Delta t},\;\Delta t=0.1\;ms$; i.e., within the compartment meshwork) where the plasma membrane is considered free standing (15). Based on Saffman-Delbrück model, $D_{micro}$ is sensitive to the size of the diffusive domain embedded in the membrane (68). Therefore, the measured fast and slow $D_{micro}$ suggest that the lipid may associate with other molecules and diffuse together as a molecular complex of two characteristic sizes and compositions. One should note that the Saffman-Delbrück model considers a free-standing membrane bounded above and below by an infinite amount of solvent with a lower viscosity than the membrane. Therefore, strictly speaking, the Saffman-Delbrück model only serves as an approximation because the plasma membrane is pinned to the cytoskeleton on the length scale of 100 nm (69), where the molecular diffusion is expected to be influenced by the attachment proteins. Previous studies have shown that membrane molecules often form clusters and move collectively (1, 2, 3,7,58,70). The diffusion of molecular complexes is governed by their interactions with the membrane structures (e.g., membrane compartments of the cytoskeleton meshwork). We find that the DSPE underwent more constrained diffusion than DOPE (with a larger ρ and a smaller L; see Table 1). This indicates that the DSPE-associated membrane clusters (most likely in the Lo and gel phases) experience a stronger confinement than the DOPE-associated membrane clusters (in the Ld phase). While our data imply an interplay between the membrane clusters and the cytoskeleton meshwork, it remains unclear how they interact and what determines their interactions. For example, are these membrane clusters pinned to the cortical actin network? Are these clusters the physical obstacles that make the picket and fence? To address these questions, simultaneous SPT of membrane molecules and superresolution imaging of the cytoskeleton meshes would be helpful.

### Cholesterol- and temperature-dependent membrane nanodomains affect phospholipid diffusion

**Figure 7:**
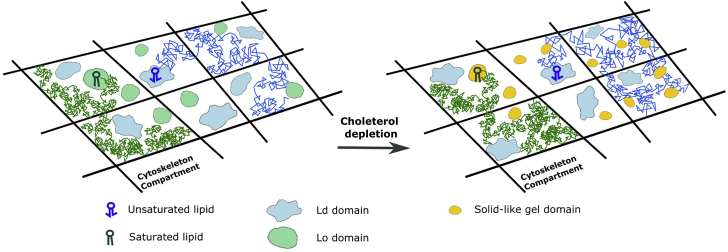
Schematic drawings of membrane organization and lipid diffusion before and after cholesterol depletion. To see this figure in color, go online.

Heterogeneous diffusion of membrane molecules is often cholesterol dependent, and their occurrences were thought to link to lipid rafts. For example, transient confinements of sphingolipids and GPI-anchored proteins were observed in the isolated cholesterol-dependent microdomains (8). Using STED-FCS, anomalous subdiffusion of sphingomyelin and GPI-anchored protein was measured at a length scale <20–40 nm by cholesterol-mediated complexes (19). Dual-mobility diffusion of cholesterol was also measured in the cell plasma membrane (28). In this work, we found that the subdiffusion of both DOPE and DSPE were also cholesterol dependent. Their diffusion became much more constrained (larger ρ and smaller L) after cholesterol depletion, which might be due to the gel-phase nanodomain formation (35,36). These nanodomains act as diffusion obstacles to DOPE and lead to a more confined diffusion of DOPE. On the other hand, DSPE could partition in the gel-phase nanodomains, whose diffusion also became more restricted compared with those in Lo phase (see Fig. 7 for a schematic diagram).

As DSPE is a probe lipid for Lo/gel phases, it is particularly interesting to examine how its diffusion was modulated by cholesterol. It is worth noting that, in our data, the $D_{micro}$ of M_fast_ of DSPE increases significantly by cholesterol depletion (from 0.86 μm^2^/s to 1.23 μm^2^/s). The significant enhancement of $D_{micro}$ for DSPE implies that there might be very small gel-phase nanodomains in the cholesterol-depleted membrane compared with the Lo nanodomains in the normal cell membrane. The observation that M_fast_ of DSPE is highly sensitive to cholesterol concentration also implies that it could be closely connected with the cholesterol-mediated Lo phase. On the contrary, the diffusion of M_slow_ of DSPE is almost insensitive to the cholesterol depletion (0.47 μm^2^/s versus 0.52 μm^2^/s), suggesting that the slow DSPE may be embedded in a molecular cluster where cholesterol depletion has little effect on its diffusion. One possible explanation is that our treatment may not be able to remove the cholesterol from the cluster due to its dense molecular packaging (59).

We measured the same trend of more constrained diffusion by lowering the temperature from 37°C to 25°C as by cholesterol depletion. This result strongly suggests that the dependency of lipid subdiffusion on cholesterol concentration and temperature originated from the membrane phase separation at the nanoscale. While it requires further investigation to verify the exact underlying mechanisms of measured subdiffusion, the different responses of the two mobilities of DOPE and DSPE to cholesterol depletion serve as the evidence that lipid diffusion in the plasma membrane is highly heterogeneous and is sensitive to the local cholesterol concentration and membrane phases.

## Conclusion

In summary, we reported nanoscopic diffusion of single phospholipids by high-speed SPT. Our high-resolution data showed that the nanoscale diffusion was spatiotemporally heterogeneous. The transient diffusion coefficient varied considerably and its fluctuation magnitude and correlation time differed between the saturated and unsaturated lipids. Compared with the unsaturated lipid of DOPE, we observed a smaller fluctuation magnitude with a longer correlation time in the transient diffusion characteristics of the saturated lipid of DSPE. The temporally stable diffusion of DSPE is interpreted as the result of its stable partition into the membrane nanodomains of the ordered phase. In addition, dual-mobility subdiffusion was measured for the two probe lipids of DOPE and DSPE on the length scale below 100 nm. The subdiffusion can be well described with the hop diffusion model. Through quantitative data analysis, we determined the effective compartment size and confinement strength experienced by the probe lipids. Our data show that DSPE underwent more restricted diffusion than DOPE. After cholesterol depletion, both diffusion of DOPE and DSPE at the nanoscale became more heterogeneous and more confined. Lowering the temperature from 37°C to 25°C had similar effects on the nanoscale lipid diffusion. This work provides the experimental evidence that the cholesterol concentration and temperature determine the nanoscopic motions of phospholipids in the cell plasma membrane. Our data imply that the phospholipids associate with other membrane molecules through the cholesterol-dependent phase separation, and these molecules diffuse together as a molecular cluster. As a result, the compartmentalized diffusion of phospholipids is determined not only by the cytoskeleton meshwork but also by the properties of the associated membrane nanodomains. We expect this conclusion to be valid for some membrane proteins. Taken together, the membrane compartmentalization by cytoskeleton and nanodomain formation through phase separation serve as the two main mechanisms for modulating membrane dynamics at the nanoscale.

## Author contributions

C.-L.H. conceived and supervised the project. Y.-J.C. and Y.-H.L. performed the SPT experiments. C.-Y.C. analyzed the data with the model. C.-H.L. prepared and verified the probe lipids. C.-L.H. and Y.-H.L. wrote the manuscript. C.-L.H. and C.-Y.C. revised the manuscript. All authors read and approved the manuscript.

## Supporting material

Document S1. Figures S1–S10 and Table S1

Data S1. The diffusion coordinates of DSPE in the plasma membrane of PtK2 cells at 37°C (DSPE.mat) The file contains 245 trajectories of DSPE. All trajectories start at the origin. The unit is nanometer.

Data S2. The diffusion coordinates of DOPE in the plasma membrane of PtK2 cells at 37°C (DOPE.mat) The file contains 240 trajectories of DOPE. All trajectories start at the origin. The unit is nanometer.

Document S2. Article plus supporting material
