# Supplementary material for: Heterogeneous nanoscopic lipid diffusion in the live cell membrane and its dependency on cholesterol
Source: Biophys J. 2022 Jul 16;121(16):3146–61. doi: 10.1016/j.bpj.2022.07.008 (PMC9463655; doi:10.1016/j.bpj.2022.07.008)
Supplement: Document S1. Figures S1–S10 and Table S1 [file mmc1.pdf]

Biophysical Journal, Volume *121*

Supplemental information

Heterogeneous nanoscopic lipid diffusion in the live cell membrane and its dependency on cholesterol

Yu-Jo Chai, Ching-Ya Cheng, Yi-Hung Liao, Chih-Hsiang Lin, and Chia-Lung Hsieh

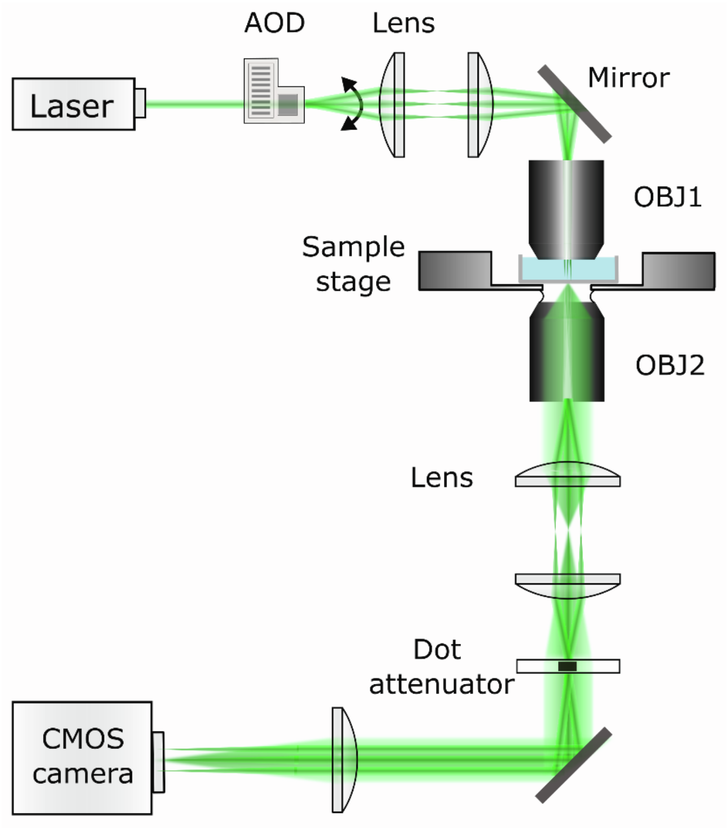

Fig. S1 Optical setup of contrast-enhanced COBRI microscopy

The contrast-enhanced COBRI microscopy used in this study. See the main text for the description of the setup.

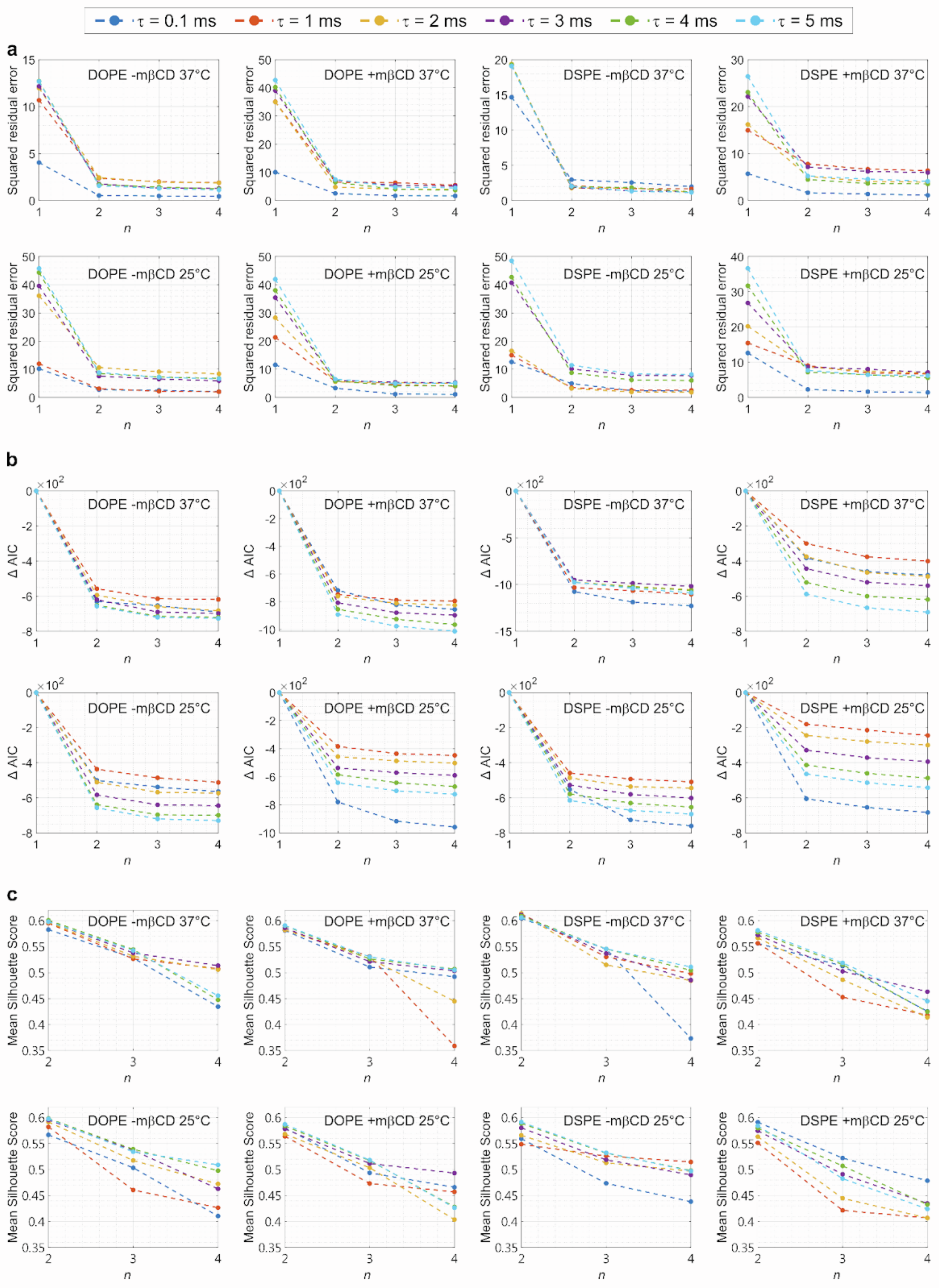
τ = 0.1 ms
τ = 1 ms
τ = 2 ms
τ = 3 ms
τ = 4 ms
τ = 5 ms
a
b
c
DOPE -mβCD 37°C
DOPE +mβCD 37°C
DSPE -mβCD 37°C
DSPE +mβCD 37°C
DOPE -mβCD 25°C
DOPE +mβCD 25°C
DSPE -mβCD 25°C
DSPE +mβCD 25°C
Squared residual error
Δ AIC
Mean Silhouette Score
n

Fig. S2 Examination of the quality of Gaussian mixture model (GMM)

We calculate the sum of squared residual error (a), the Akaike information criterion (AIC) (b), and the silhouette coefficient (c) for different numbers of components *n* (*n* = 1 to 4; note that n $\geq$ 2 for the silhouette coefficient). The squared residual error is reduced significantly when adding the second component, indicating that at least a second component is needed. The reduction of residual error becomes marginal when including the third/fourth component, suggesting that these components may not be critical and the data could be overfitted. We also calculate the AIC to estimate the error of statistical prediction and thereby the quality of the mixture model. The lower the AIC, the better the quality of the model. In our case, we do see a decrease of AIC when adding the number of populations [shown in Fig. S2. Here ΔAIC is defined as the AIC(*n* components) − AIC(1 component)]. In theory, AIC deals with the trade-off between the goodness of the fitting and the simplicity of the model, so the best model should give the lowest AIC. In practice, however, due to the inevitable errors in the data (systematic noise, limited dataset, etc.), 'elbow method' is commonly used to determine the optimal number of Gaussian components (1). The elbow method identifies the number of components where the AIC gradient is changed significantly, which suggests two populations in our data. Finally, we calculate the Silhouette coefficient for different numbers of components, a common method to determine the optimal number of components for the mixture model (2). The more the Silhouette coefficient is near to one, the better the model is. Once again, the Silhouette coefficient analysis shows that the two-component model is the best model compared to the three- and four-component models. Based on the above examination, we conclude that the histogram of D_{app} is best described by a mixture of two mobilities.

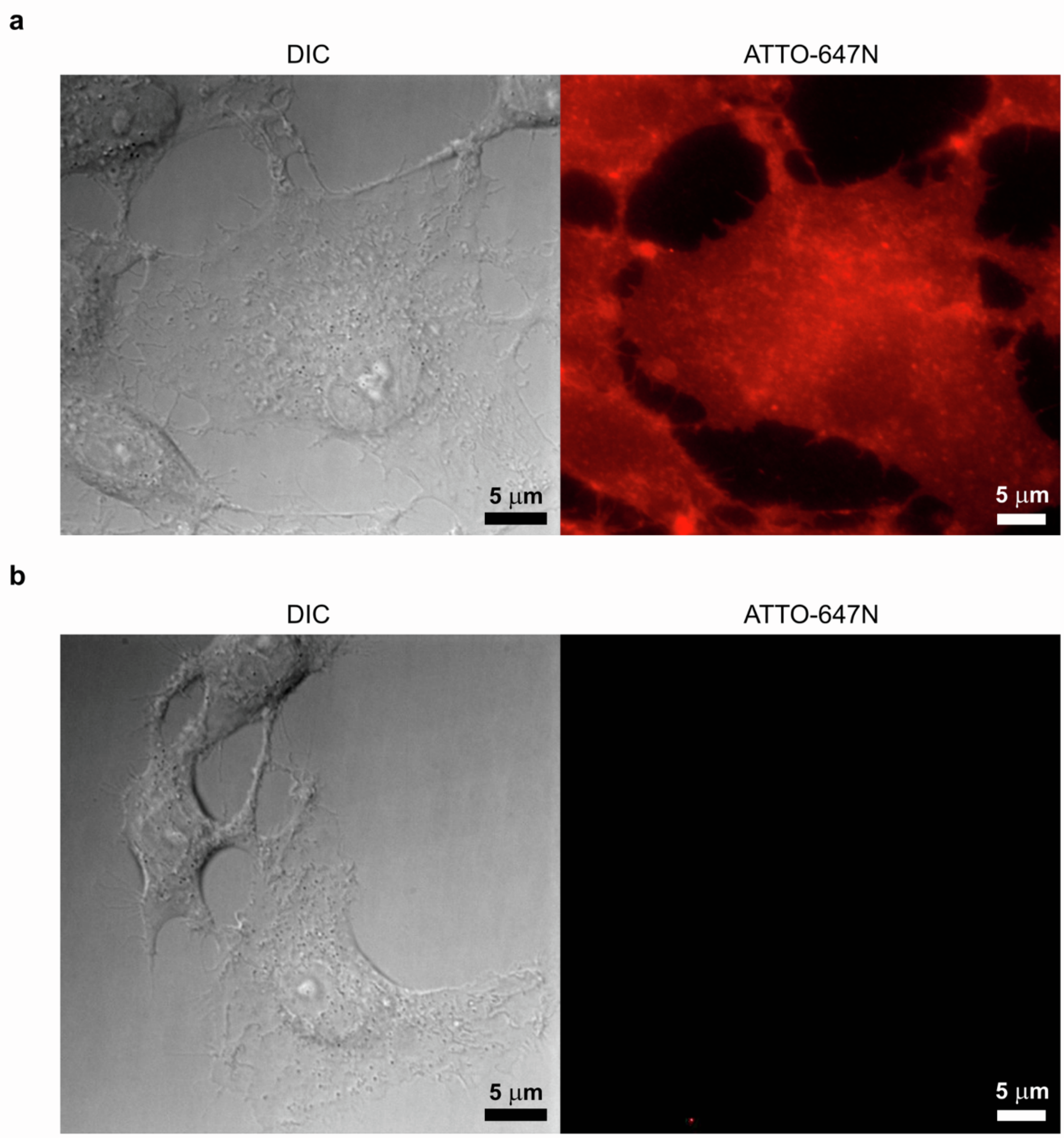

Fig. S3 Labeling the biotinylated probe lipids on cell membrane by ATTO-647N conjugated streptavidin

Introduction of biotinylated probe lipids to the cell plasma membrane is confirmed by successful labeling of ATTO647N-conjugated streptavidin on the probe lipids (DOPE-PEG-biotin), shown in (a). The labeling of ATTO647N-conjugates streptavidin is specific because no labeling is observed in the cells without introducing the biotinylated probe lipids, shown in (b).

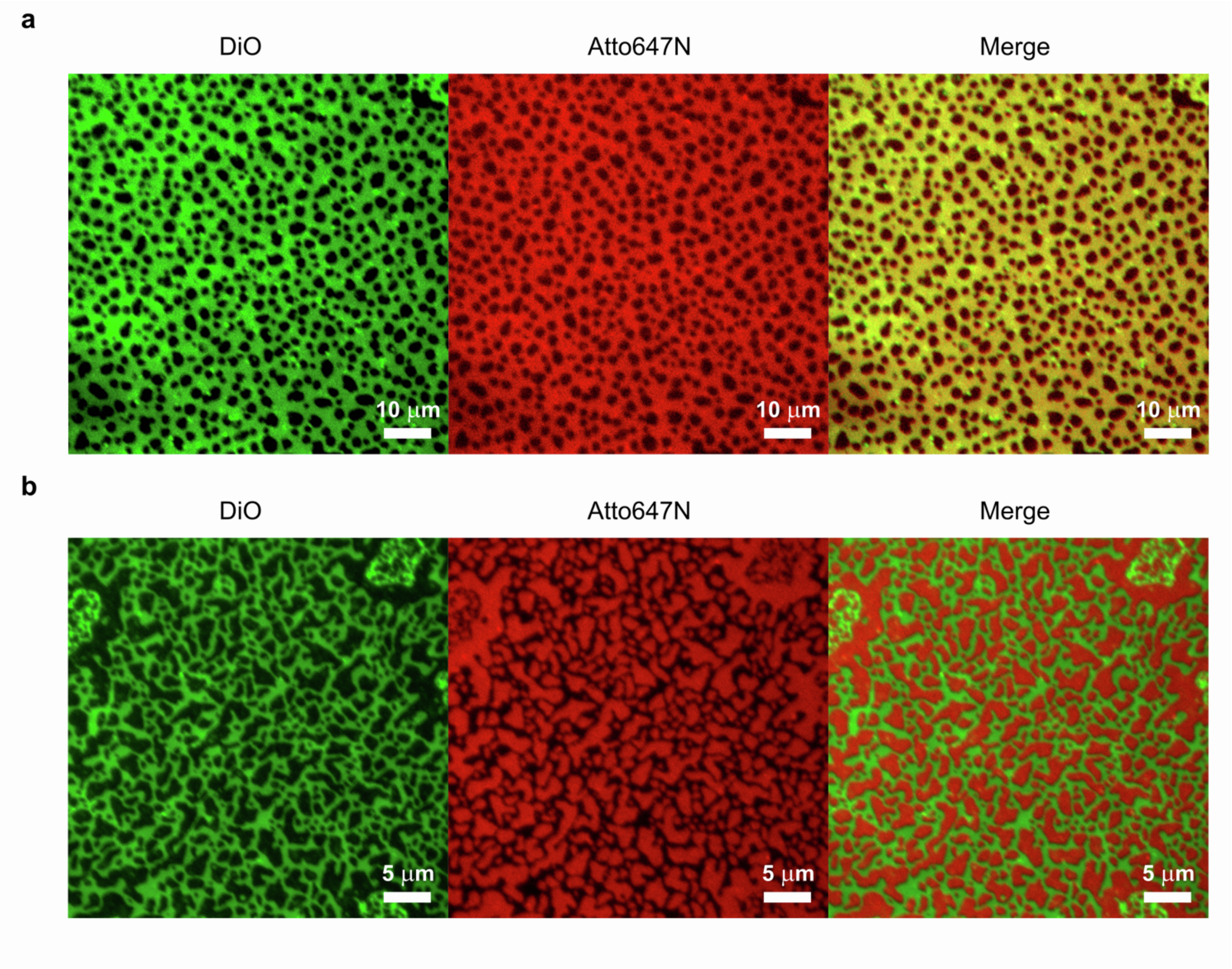

Fig. S4 Partition of biotinylated probe lipids in Ld/Lo coexisting SLBs

The preferential Ld/Lo partitioning of DOPE-PEG-biotin and DSPE-PEG-biotin is verified in the model membranes. By mixing DPPC:DiphyPC:cholesterol at 40:40:20 molar ratio, Ld/Lo phase coexisting supported lipid bilayer forms on the mica substrate (see the main text for the preparation details). The biotinylated probe lipid (~1.25 mol% of DOPE or DSPE) is added to the membrane. Meanwhile, a trace amount of lipophilic fluorescent dye DiO is added to mark the region of Ld phase. Thus, in the DiO map (green), the bright regions correspond to the Ld phase, whereas the dark regions correspond to the Lo phase. The biotinylated probe lipids are labeled by ATTO647N-conjugated streptavidin whose location is imaged by fluorescence microscopy (middle red images). Our data show that biotinylated DOPE colocalizes with the Ld regions (a), and biotinylated DSPE colocalizes with the Lo regions (b).

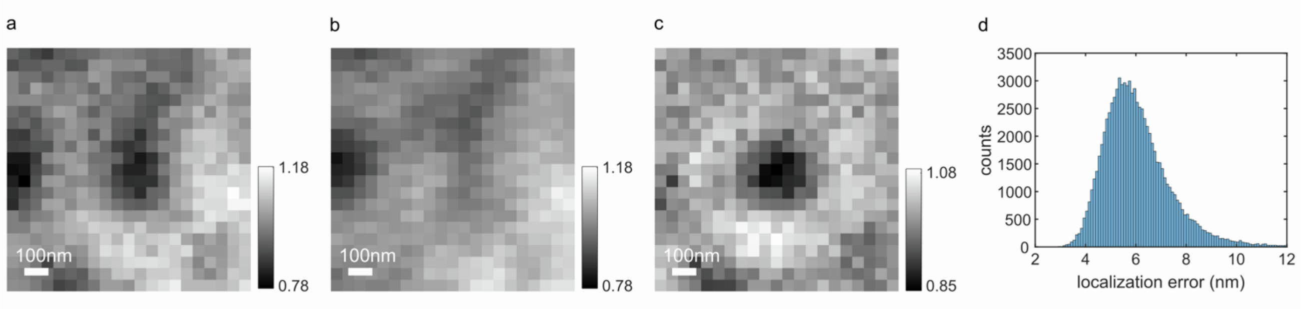

Fig. S5 Background estimation and correction for high-precision nanoparticle localization

(a) Raw image of a nanoparticle on the cell membrane. (b) Estimated cell background (see main text for the estimation algorithm). (c) Background removed image of the nanoparticle. (d) Histogram of the localization precision of the nanoparticle.

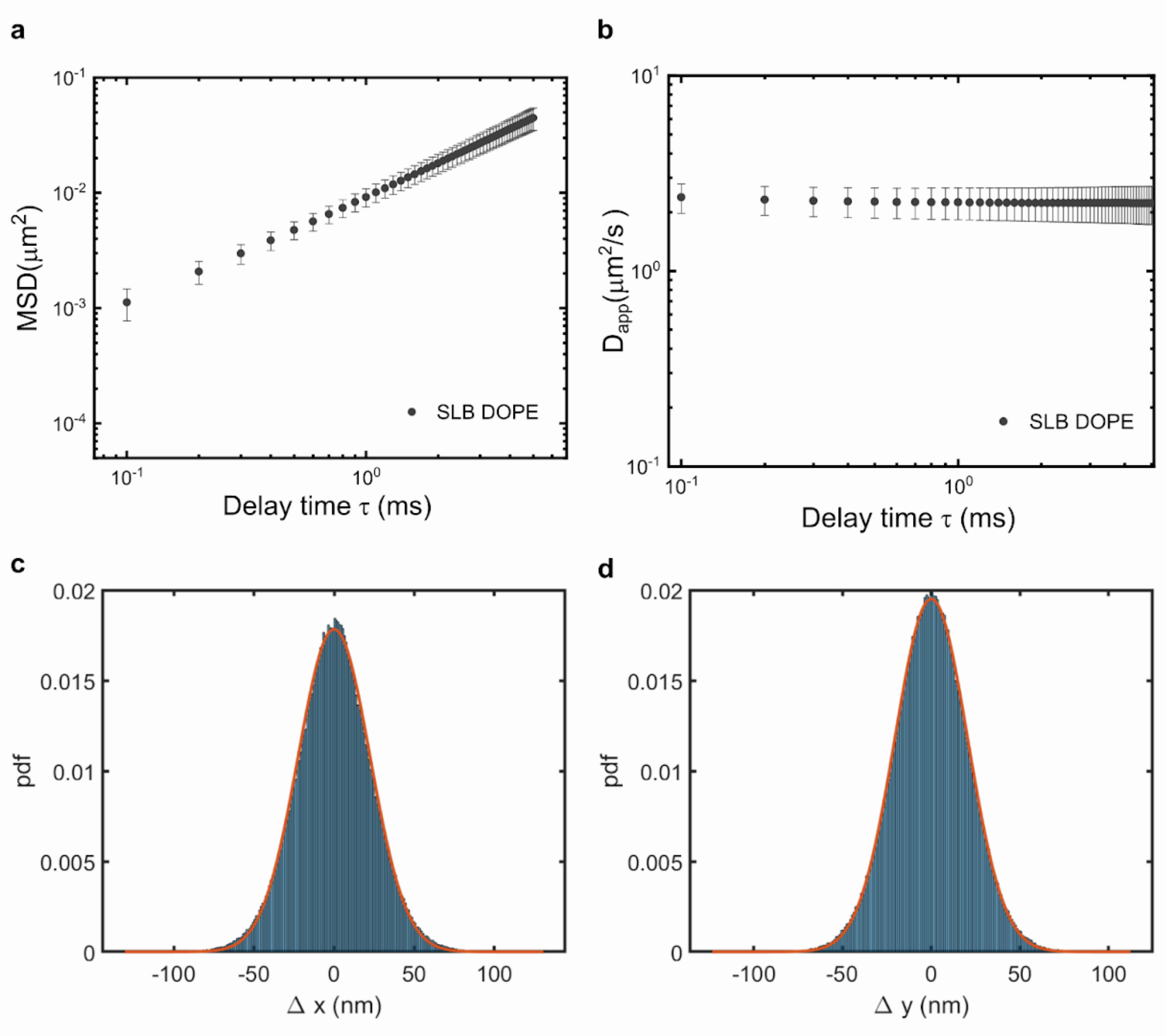

Fig. S6 Free diffusion is detected by SPT in the homogeneous model membrane of the Ld phase

We perform high-speed SPT of biotinylated DOPE in the supported lipid bilayer (SLB) consisting of DOPC at room temperature. Free diffusion is observed in all time scales ranging from 0.1 ms to 5 ms, producing a linear dependency of MSD on delay time (a), and a constant D_{app} over different delay time (b). The probability density function (pdf) of the lateral displacement at the 0.1 ms time interval exhibits a Gaussian distribution, displayed in (c) and (d) for the x and y directions, respectively. Note that the histograms in (c) and (d) are experimental data, whereas the red curves are the fitted Gaussian functions.

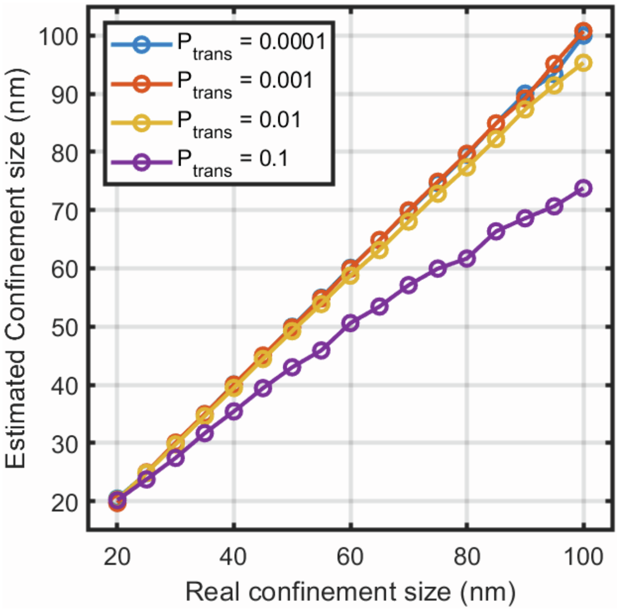

Fig. S7 Accuracy of compartment size estimation by hop diffusion analysis

We systematically simulate hop diffusion trajectories in periodic diffusion barriers, where the compartment size L and the transmission probability (corresponding to the confinement strength ρ) are freely adjustable parameters. The D_{micro} is set as 0.6 μm^2/s, approximately the average value of those of M_{fast} and M_{slow}. For each condition, 1000 trajectories (each of which consists of 1000 steps) are simulated, and their ensemble D_{app} is fitted with the analytical approximation of hop diffusion model (Eq. (5) in the main text). The estimated compartment size L is plotted aginst its real size, ranging from 20 nm to 100 nm. We note that the estimation is accurate when the transmission probability is less than 0.001. For the weakly confined cases (where the transmission probability is > 0.01), the L is systematically underestimated. This is because in the weakly confined scenarios, the particle does not explore the whole compartment area before hopping to the adjacent zone, leading to a smaller estimated compartment size.

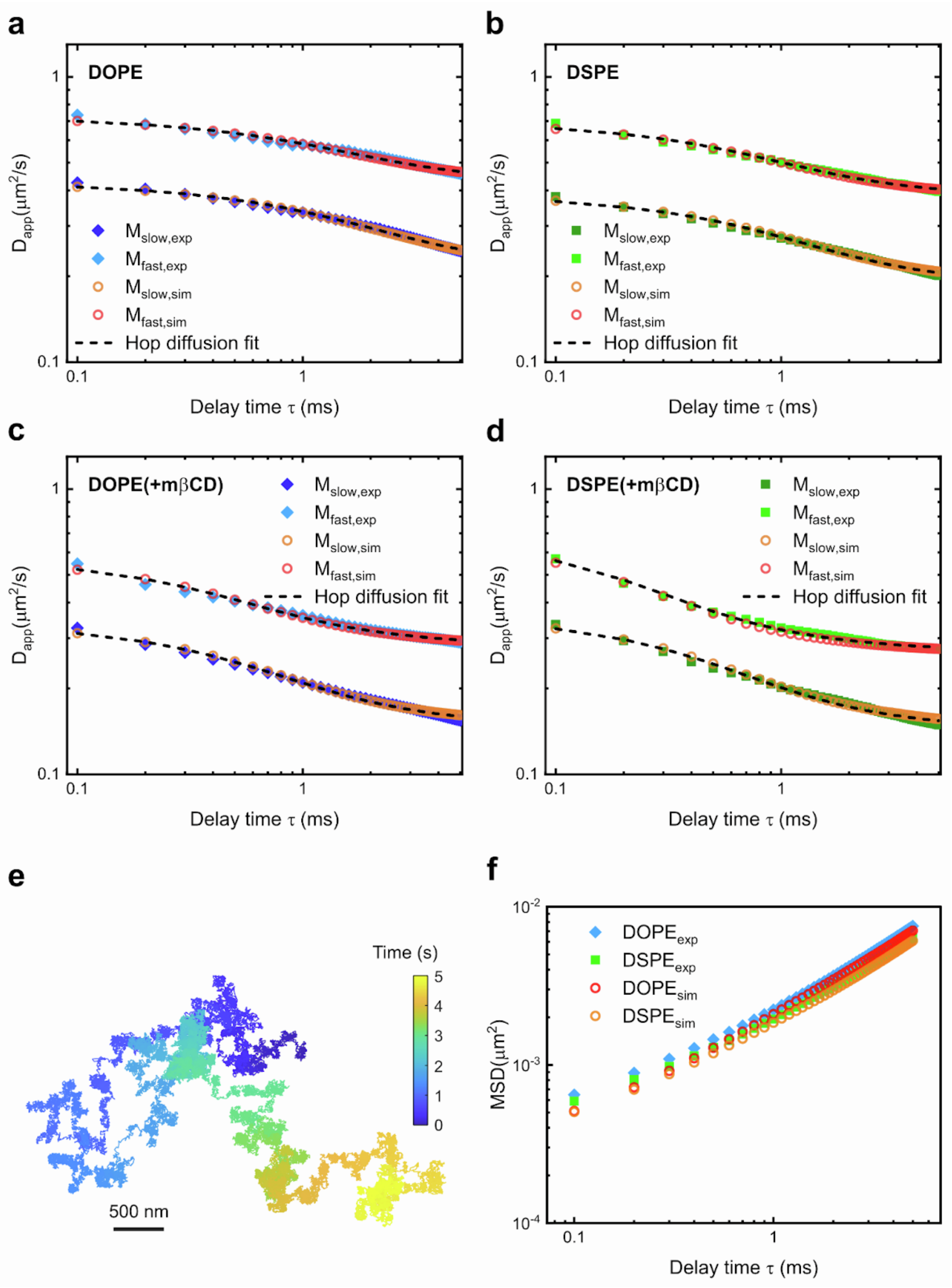

Fig. S8 The simulated hop diffusion trajectories agree well with the experimental data.

We simulate hop diffusion trajectories and find good agreements with the experimental results. (a)-(d) The D_{app} as a function of delay time of the simulated hop diffusion and the experimental data measured at 37°C. Simulated trajectories are created separately for each experimental condition (DOPE/DSPE, with/without cholesterol depletion) and for each mobility (M_{fast}/M_{slow}). In all cases, the simulated results agree well with the experimental data, indicating that hop diffusion is sufficient for reproducing the subdiffusion characteristics over the timescale of 0.1 ms to 5 ms. The parameters used for the simulation are summarized in Table S1. (e) A simulated dual-mobility diffusion trajectory

of DOPE in the cell plasma membrane. By randomly mixing and connecting the simulated hop diffusion trajectories of M_{fast} and M_{slow} at a population ratio of 40:60 (as indicated in the experimental data Fig. 3e), we generate the dual-mobility diffusion trajectory that mimics DOPE diffusion in the cell plasma membrane. (f) The MSD analysis of the simulated dual-mobility diffusion of DOPE and DSPE. We simulate the dual-mobility diffusion trajectories for DOPE and DSPE and calculate their MSDs. The simulated results are highly correlated to the experimental data, both showing anomalous subdiffusion with an anomalous exponent of 0.8 approximately. It supports that the dual-mobility hop diffusion can reproduce the lipid subdiffusion in the cell plasma membrane over the timescales of 0.1 ms to 5 ms.

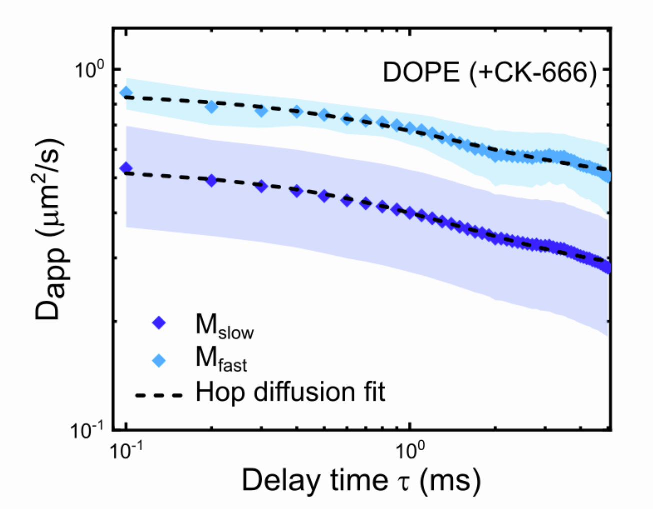

Fig. S9 Inhibition of actin depolymerization by CK-666 results in an increased compartment size

SPT measurements were performed on DOPE in the CK-666 treated cells. By analyzing the dual-mobility subdiffusion data with the hop diffusion model, we determine a compartment size of 82 ± 3 (66 ± 1) nm for the M_{fast} (M_{slow}) of DOPE, which is larger than that measured in the normal cells. It indicates that the cortical actin meshwork is responsible for the confinement in the hop diffusion model.

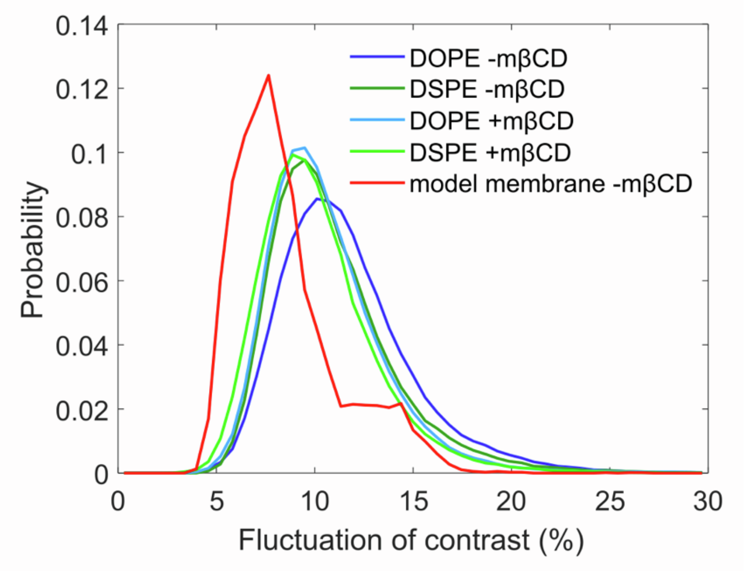

Fig. S10 Estimation of membrane flatness based on the particle contrast fluctuation

To verify whether the nanoscopic membrane topology is altered in the treated cells, we look into the SPT image data. Under our interference microscopy, the optical contrast of the particle is a function of its axial position (see detailed characterization in our previous publication (3)). Thus, we can estimate the vertical displacement of the particle, and thus the membrane roughness, from the change of optical contrast. We determine the particle contrast in every optical image and then analyze its temporal fluctuation. When the membrane is perfectly flat, we expect a constant optical contrast. When the membrane is rough, the particle contrast varies in time. The detection sensitivity is determined by the precision of measuring the particle contrast that is ultimately set by the measurement noise. To quantitate the contrast fluctuation, we calculate the standard deviation (std) of particle contrast of all trajectory segments with a time window of 5 ms (50 steps), corresponding to an average diffusion length of ~125 nm ($\sqrt{4D_{micro}\Delta t} = \sqrt{4 \times 0.8\mu m^2/s \times 5ms} = 126\,nm$). Fig. S10 plots the fluctuation of contrast for all experimental conditions. Here the fluctuation of contrast is defined as the ratio between the std of particle contrast and the average particle contrast. We measure the smallest fluctuation measured in the model membrane. Meanwhile, we find the statistically similar fluctuations for all other conditions (DOPE/DSPE, with/without cholesterol depletion). Thus, we conclude that no measurable change in the cell membrane topology below 100 nm is detected in the treated cells.

Table S1 Parameters used in the simulation presented in Fig. S8.

		Mobility	Compartment Size L (nm)	Transmission Probability p_{trans}	Microscopic diffusion coeff. D_{micro} (μm^2/s)
-mβCD	DOPE	Fast	106	0.13	0.83
		Slow	86	0.09	0.49
	DSPE	Fast	79	0.13	0.86
		Slow	62	0.11	0.47
+mβCD	DOPE	Fast	54	0.13	0.81
		Slow	47	0.10	0.46
	DSPE	Fast	45	0.09	1.44
		Slow	43	0.10	0.52

Reference

1. Shi, C., B. Wei, S. Wei, W. Wang, H. Liu, and J. Liu. 2021. A quantitative discriminant method of elbow point for the optimal number of clusters in clustering algorithm. *EURASIP Journal on Wireless Communications and Networking*. 2021(1):31.
2. Rousseeuw, P. J. 1987. Silhouettes: A graphical aid to the interpretation and validation of cluster analysis. *Journal of Computational and Applied Mathematics*. 20:53-65.
3. Huang, Y.-F., G.-Y. Zhuo, C.-Y. Chou, C.-H. Lin, W. Chang, and C.-L. Hsieh. 2017. Coherent brightfield microscopy provides the spatiotemporal resolution to study early stage viral infection in live cells. *ACS Nano*. 11(3):2575-2585
